# Biometric recognition of newborns and infants by non-contact fingerprinting: lessons learned

**DOI:** 10.12688/gatesopenres.12914.2

**Published:** 2019-11-05

**Authors:** Steven Saggese, Yunting Zhao, Tom Kalisky, Courtney Avery, Deborah Forster, Lilia Edith Duarte-Vera, Lucila Alejandra Almada-Salazar, Daniel Perales-Gonzalez, Alexandra Hubenko, Michael Kleeman, Enrique Chacon-Cruz, Eliah Aronoff-Spencer

**Affiliations:** 1 California Institute for Telecommunications and Information Technology, University of California, San Diego, La Jolla, Ca, 92093, USA; 2 Campus ECISALUD, Universidad Autonoma de Baja-California, Tijuana, Baja-California, Mexico; 3 School of Global Policy and Strategy, University of California San Diego Medical Center, La Jolla, Ca, 92093, USA; 4 Pediatric Infectious Diseases, General Hospital of Tijuana, Tijuana, Baja-California, Mexico; 5 Division of Infectious Diseases and Global Public Health, University of California San Diego Medical Center, La Jolla, Ca, 92093, USA; 6 The Design Lab, University of California, San Diego, La Jolla, Ca, 92093, USA

**Keywords:** infant fingerprinting, infant biometrics, non-contact fingerprinting, infant identification, newborn identification

## Abstract

Despite years of effort, reliable biometric identification of newborns and young children has remained elusive. In this paper, we review the importance of trusted identification methods, the biometric landscape for infants and adults, barriers and success stories, and we discuss specific failure modes particular to young children. We then describe our approach to infant identification using non-contact optical imaging of fingerprints. We detail our technology development history, including Human-Centered Design methods, various iterations of our platform, and how these iterations addressed failure modes in the identification process. We close with a brief description of our clinical trial of newborns and infants at an urban hospital in Mexico and report preliminary results that show high accuracy, with matching rates consistent with acceptable field-performance for reliable biometric identification in large populations.

## Disclaimer

The views expressed in this article are those of the author(s). Publication in Gates Open Research does not imply endorsement by the Gates Foundation.

## Introduction

Globally over 1 billion people lack legal identification and almost half of them are infants and children ^1^. To address the need, United Nations Sustainable Development Goal 16.9 calls to provide legal identity for all, including free birth registrations by 2030 ^2^. Today one of the primary barriers to fulfilling SDG 16.9 has been the lack of universal biometric technology able to reliably identify newborns, young children and even at times adults ^3^. There have been numerous attempts to utilize standard fingerprint technologies with infants with limited success; and while new technologies have been developed to address the problem, improvements have been limited to children over 6 months of age ^4^.

We investigated an array of biometric methods for infant identification. These included eye scanning, palm vein scanning, ear and face recognition, and finger and palm-based methods. To date none of these has shown to be reliable for newborn and very young infant enrollment, and only fingerprinting has shown promise in terms of universality, acceptability, persistence over time from birth ^5^ and interoperability across acquisition methods ^6– 10^. We hypothesized that the malleability of infant skin coupled with grasping and other infant reflexes were leading to deformation of the fingerprint by current biometric scanner platens themselves, which explains why even higher resolution platforms have still failed with children under six months of age. To test this hypothesis, and potentially develop a reliable infant biometric, we developed a modular biometric prototyping platform that provided a common imaging back-end to be coupled with various front-ends allowing a wide variety of infant-device-practitioner interactions. Using this method, we compared two contact-based approaches, frustrated total internal reflection (FTIR), non-FTIR direct imaging, and non-contact imaging approach with multiple interaction designs ^11^. Based on testing in laboratory and clinical settings we concluded that a non-contact imaging method was the best for newborns and infants. 

This report is meant to accompany subsequent publications detailing the performance of our non-contact technology in clinical testing. Here we report our design strategy, detailed analysis of failure modes for infant fingerprinting, and key insights and requirements that can instruct development of reliable and usable infant-centric biometrics.

## Methods

### Approach

We employed Human-centered Design principles of early stakeholder engagement and co-design, problem reframing, and agile prototyping, to rethink the infant ID problem ^12– 14^. We worked with children from birth through 18 months, along with parents, caregivers, nurses, doctors, health officials and vaccinators, to observe infant behavior and caregiver interaction through many stages of iteration of both contact and non-contact system designs. Through this process, we accumulated numerous lessons-learned, resulting in optimized procedures and a system design that enabled enrollment and scanning even in demanding field conditions. This report highlights key issues we encountered, and the steps taken to resolve them to achieve a robust infant biometric system.

In order to develop an effective infant fingerprint platform, it is critical to test in realistic environments on infants of all ages. Infants are simply not smaller adults, they have both physical and behavioral differences that cannot be readily modelled, resulting in the need for real-world testing. We established multiple field sites which allowed the team to develop and test hardware prototypes quickly and provide feedback for an iterative hardware design process. We established IRB approved pilots locally at Rady Children’s Hospital and Jacobs Medical Center at UCSD and an international study at Tijuana General Hospital to enroll newborns and older infants receiving vaccinations. These studies have provided critical insight into the requirements for the infant fingerprint device, as well as requirements for production technology, workflow and system integration. The following sections highlight the hardware development, testing process, and lessons learned for effective collection of infant fingerprints.

### Early lessons and system requirements

**Figure 1.**
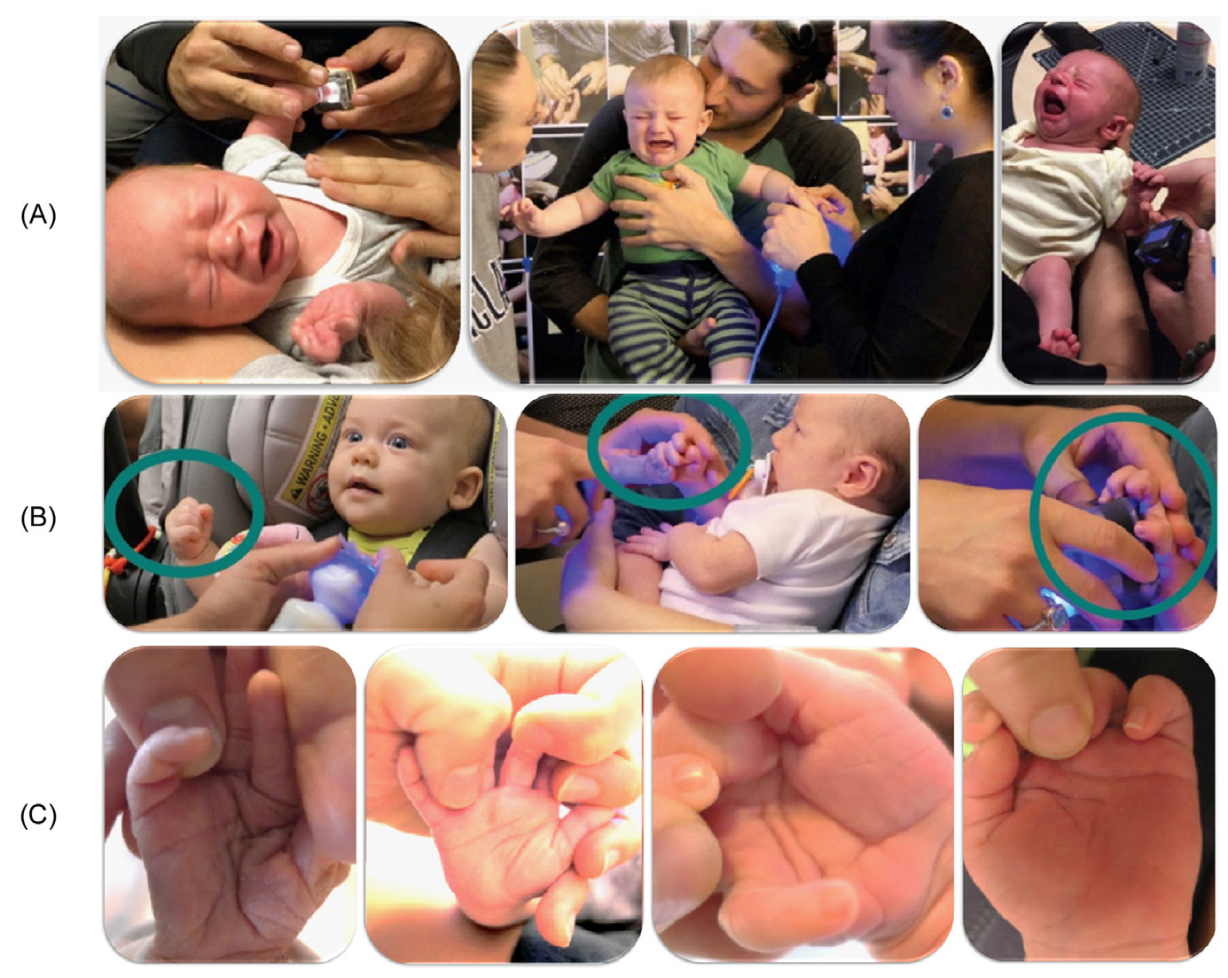
Interaction design must account for infant reflexes and behaviors. Written informed consent was obtained from parents/guardians for the publication of all images.

A major factor affecting the ability to obtain a good infant fingerprint image is the behavior and interaction between child and practitioner. Infants are “uncooperative”, and the device design and collection protocol must be consistent with how babies, and often caregivers, behave, not how technology functions best. As Figure 1(a) shows, infants cry, fuss and cannot be asked to participate. Under normal circumstances (when fingerprinting or taking an eye scan with an adult, for example) the subject can interact with the device in a predictable and deliberate fashion. 

Current technologies expect the subject to properly place the finger, hold still, and move to other positions based on feedback from the device or a practitioner. Infants, on the other hand, need to be helped through the process and may exhibit behaviors that are not helpful, such finger curling and the palmar grasp reflex, as shown in Figure 1(b). One of the first design considerations was whether to simply hold the infant’s hand in the proper position and use a point-and-shoot camera or smart phone to obtain images. As the images in Figure 1(c) elucidate, it is difficult to reproducibly obtain images with an infant in this manner. Holding their hands open to collect finger or palm prints requires multiple people and the variability in magnification, lighting, and field of view of the images taken by a practitioner pointing a camera at the infant’s hand can be too great, even with automated image processing and machine learning.

Another key factor is that newborns exhibit skin characteristics unique to the first days and weeks of life ^15, 16^. For example, newborns are born covered in various protective fluids that are wiped off shortly after birth, and it is normal for a newborn’s outer layer of skin to flake or peel within the first weeks of life. Likewise, physiological changes that increase skin integrity, including keratinization, collagen development, and component cross-linking continue to develop after birth, and in some cases may not stabilize sufficiently for contact-based fingerprinting until over one year of age.

**Figure 2.**
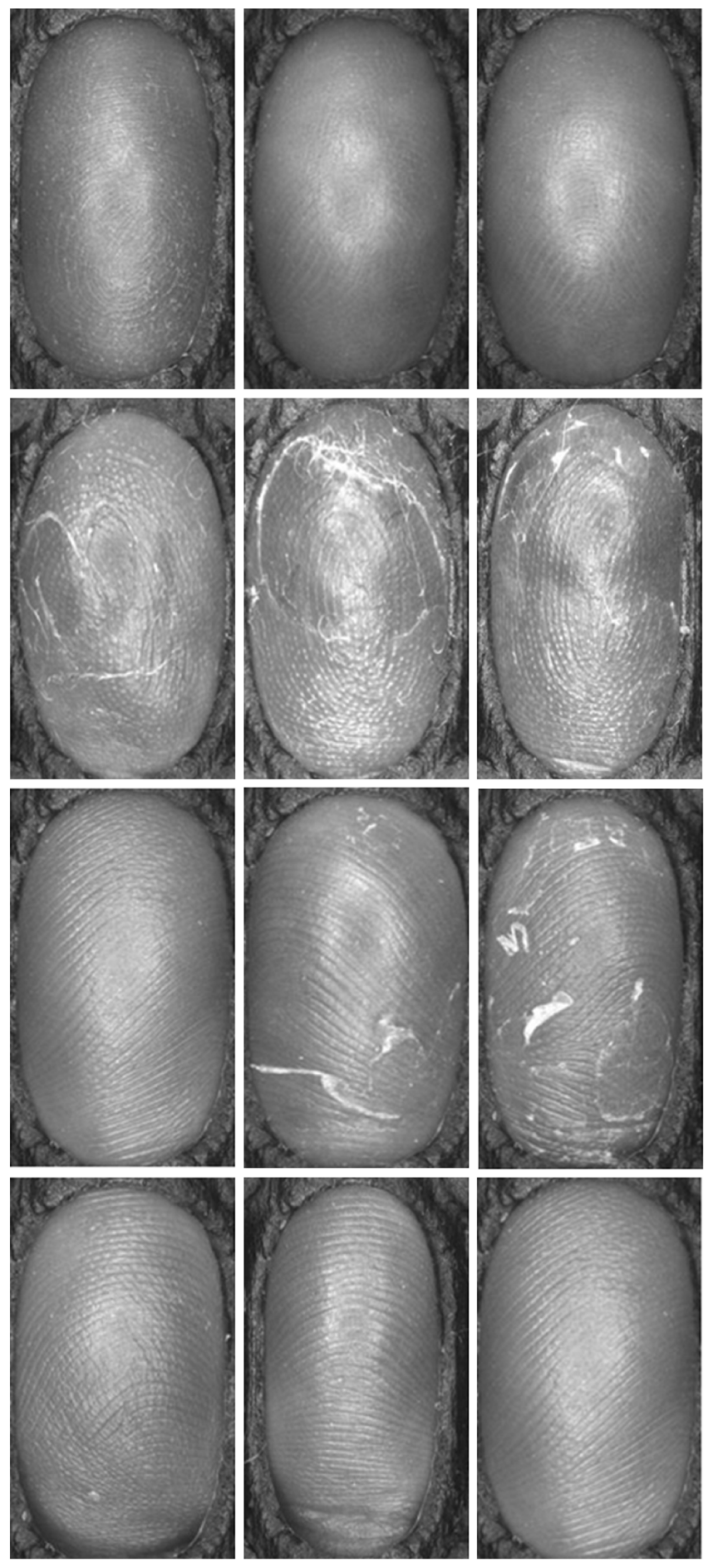
Newborn skin peeling issues: (Top to bottom) pre-peeling, peeling, partial peeling and post-peeling.

As shown in Figure 2, we see four stages that will impact performance in both contact and non-contact use cases. Starting at the top, we see a pre-peeling stage, where the infant’s fingers are shiny (highly reflective) and there is a dead layer of skin masking the print. Next, we see the skin dramatically peeling from the fingers. Again, the peeling skin will mask the underlying print and impact the automatic image processing algorithms that are used to evaluate the fingerprint pattern. The third row shows an infant that is partially peeling, and the bottom panel shows how the prints emerge post-peeling, which occurs at about one month. 

**Figure 3.**
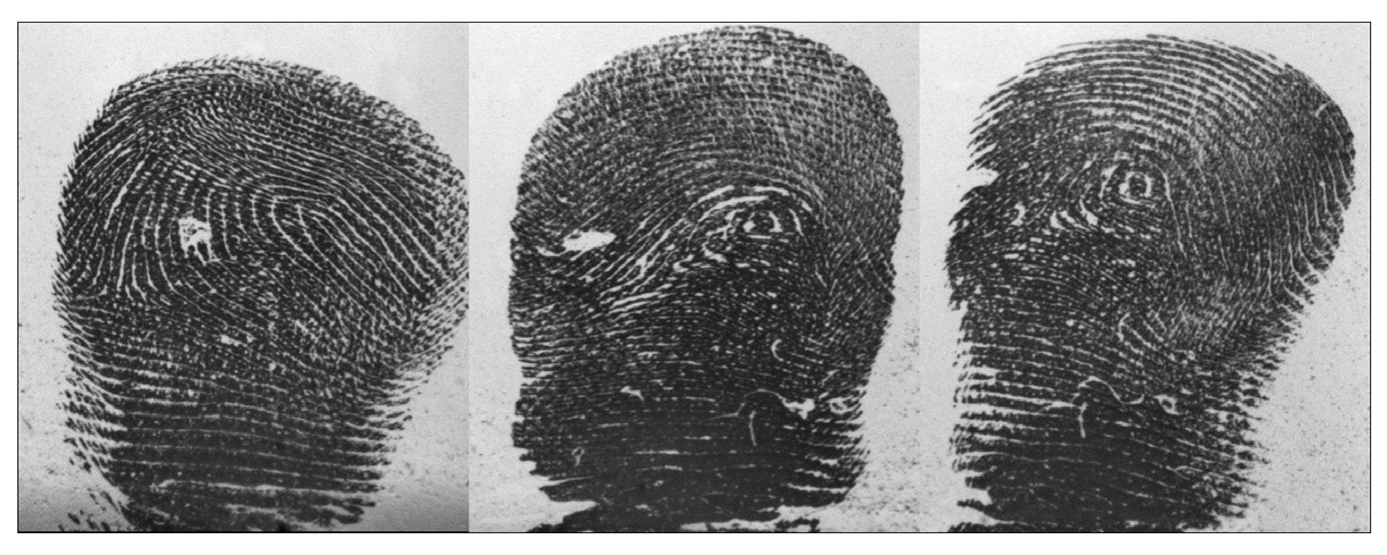
Sequential FTIR images of a single infant finger showing collapse of features and distortion of minutiae position.

Another issue observed is that infant skin surface is much softer and more pliable than that of an adult. This apparent malleability was a major factor in our decision to use a non-contact method of imaging. As an example, when using an FTIR scanner, the infant fingerprint will flatten against the surface and the ridges will “squish” leading to fusion of features when imaged. Adjacent ridges essentially merge together reducing the air gap that allows the light to reflect to create contrast between the ridges and valleys. An additional concern relates to the interaction of the infant finger with the device. An adult can gently place their finger onto the surface of the device and keep contact with little distortion. When the infant’s finger is placed onto the glass platen, the grasp reflex will cause the finger to react inconsistently. Typically, a single portion of the finger will contact first, then the rest will contact as the finger is put into place. Invariably, the finger does not go down the same way each time and the print is distorted due to the skin pliability. Figure 3 shows a series of images of the same finger on three consecutive collections within seconds of each other using a high resolution FTIR contact device. What we see is poor contrast between some of the ridges due to the softness of the skin and the relative positions of the minutia are quite variable, which is a significant barrier for infant fingerprinting using this method.

**Figure 4.**
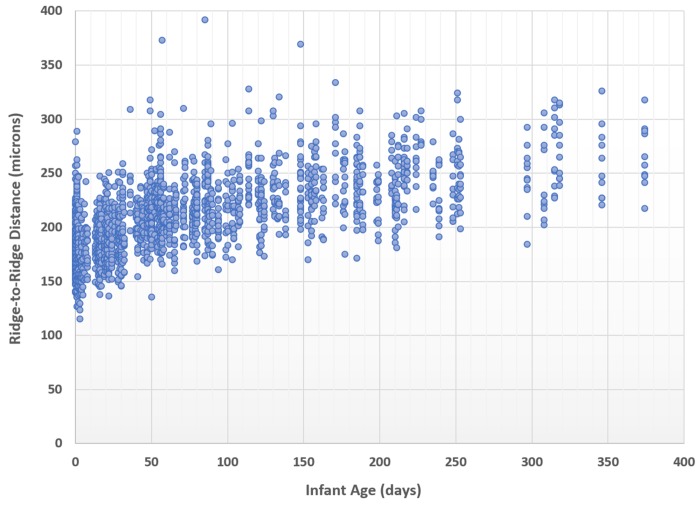
Infant fingerprint feature size vs age.

**Figure 5.**
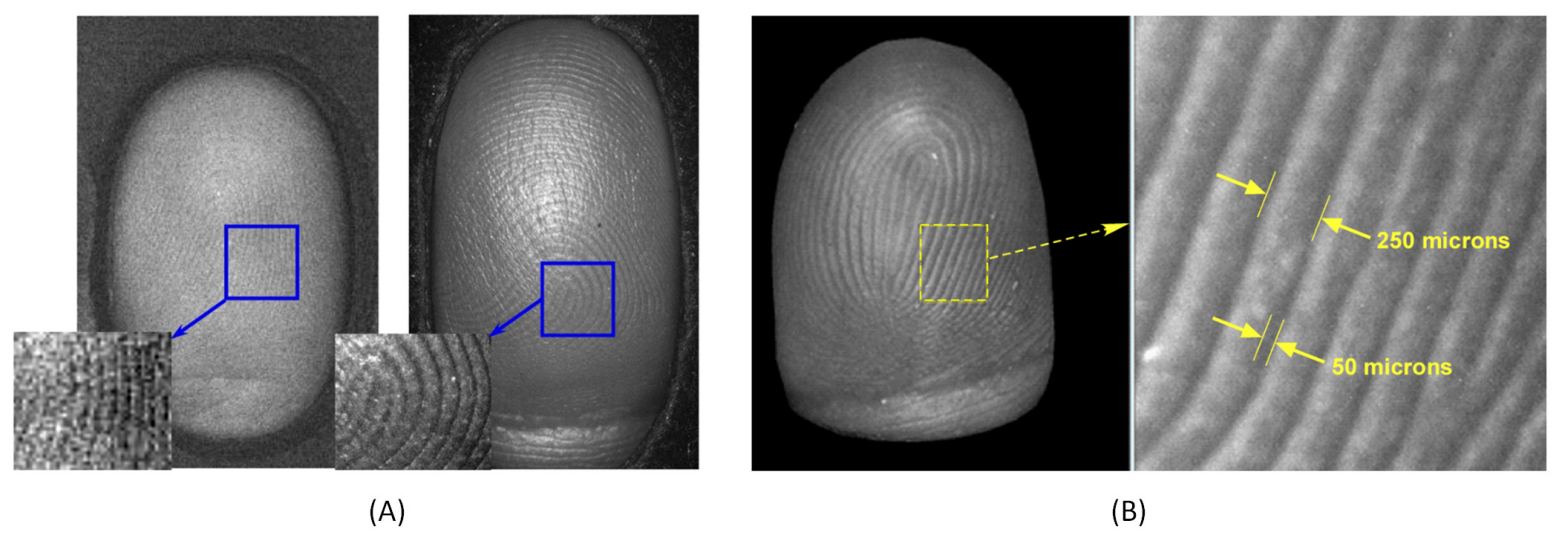
( **a**) Image comparison of infant finger taken at 1500 ppi (left) and 3400 ppi (right) and ( **b**) variations in ridge and valley sizes.

Finally, infants are smaller than adults. A person’s unique fingerprints are formed prior to birth and the ridge/valley features that exist on the adult fingerprint are all present on the much smaller sized newborn finger. The ridge-to-ridge feature size of a newborn fingerprint can be as small as 125 microns, as shown in Figure 4, from our data on hundreds of newborns and infants. This is much smaller than the 450-micron distance for a typical adult ^17^. A standard scanner operating at 500 PPI would only allocate 2.5 camera pixels to resolve the newborns ridge/valley feature. As other researchers have tried before us, we increased the resolution of the imager to ~1500 PPI to account for the smaller feature size of infants. After collecting data on a number of infants at this resolution, we determined that even higher resolution was still needed. There are several reasons for this: the first is unique to non-contact imaging where a 2-D picture is rendered from a finger that has 3-D curvature. One of the advantages of the contact-based method is that the edges of the fingerprint can be put in contact with the glass. When this occurs, the print on the side of the finger is brought to the image plane and the feature size dimension is retained. The camera “sees” the same feature size whether it is in the middle of the finger or on the edge. For a non-contact imager, however, the feature size will be largest in the middle of the finger perpendicular to the camera, but the ridge-to-ridge distance will decrease (essentially to zero) as you move around to the side of the finger as seen from the direction of the camera. Thus, to continue to resolve the print features as the finger starts to curve away from the camera, we need to resolve smaller and smaller features. Figure 5(a) shows an example of an infant image at 1500 PPI and at 3600 PPI and the resolving power of the 3600 PPI provides good contrast of features across a large area of the fingerprint. 

After collecting many hundreds of prints, we determined that the peak-to-peak measurement that reports the feature size does not tell the entire story. Figure 5(b) shows a close-up view of an infant’s fingerprint. The left panel shows the entire finger and the right panel shows a view within the yellow box. What is plain to see is that the valley thickness is not equal to the ridge thickness. For an infant with a ridge-to-ridge feature size of 250 microns, for example, the ridge thickness can be 200 microns wide and the valley only 50 microns. Thus, it is incorrect to say that there is a feature size of 250 microns that needs to be resolved. On the contrary, we need to resolve the 50-micron valley. This ratio of peak-to-valley thickness varies from person to person, but for infants we observe the peak is often 4x-5x wider. As a result, we increased our resolution up to the ~3400 PPI which places about ~7 pixels across the valley of the print.

### Platform design

The goal was to design a dedicated, handheld device with features that reduce the impact of infant-related failure modes in order to obtain reproducible, high contrast fingerprint images. Given the need to rapidly iterate and test in real circumstances, we used a modular, easily reconfigurable hardware design. When developing any optical system, there are some key design elements that need to be optimized for a specific application. For this effort, we specifically addressed the following:

- Illumination – for fingerprint imaging, the goal is to obtain images with high contrast between the ridges and valleys so that minutae can be readily interpreted with automated image processing. The illumination color, polarization, and angle of incidence all impact the contrast and fingerprint image quality.
- Imaging system – the imaging system design starts with the selection of the imaging chip and an appropriate lens system to resolve the small infant features with a field of view that covers the entire finger of an infant (and that of an adult). In addition, the depth of field needs to be sufficient to create sharpness across the curvature of the finger.
- Finger alignment – as discussed, infants move around in an uncooperative fashion. The system design must allow for reproducible placement of fingers such that the fingerprint is minimally distorted, often with vastly different sizes.

**Figure 6.**
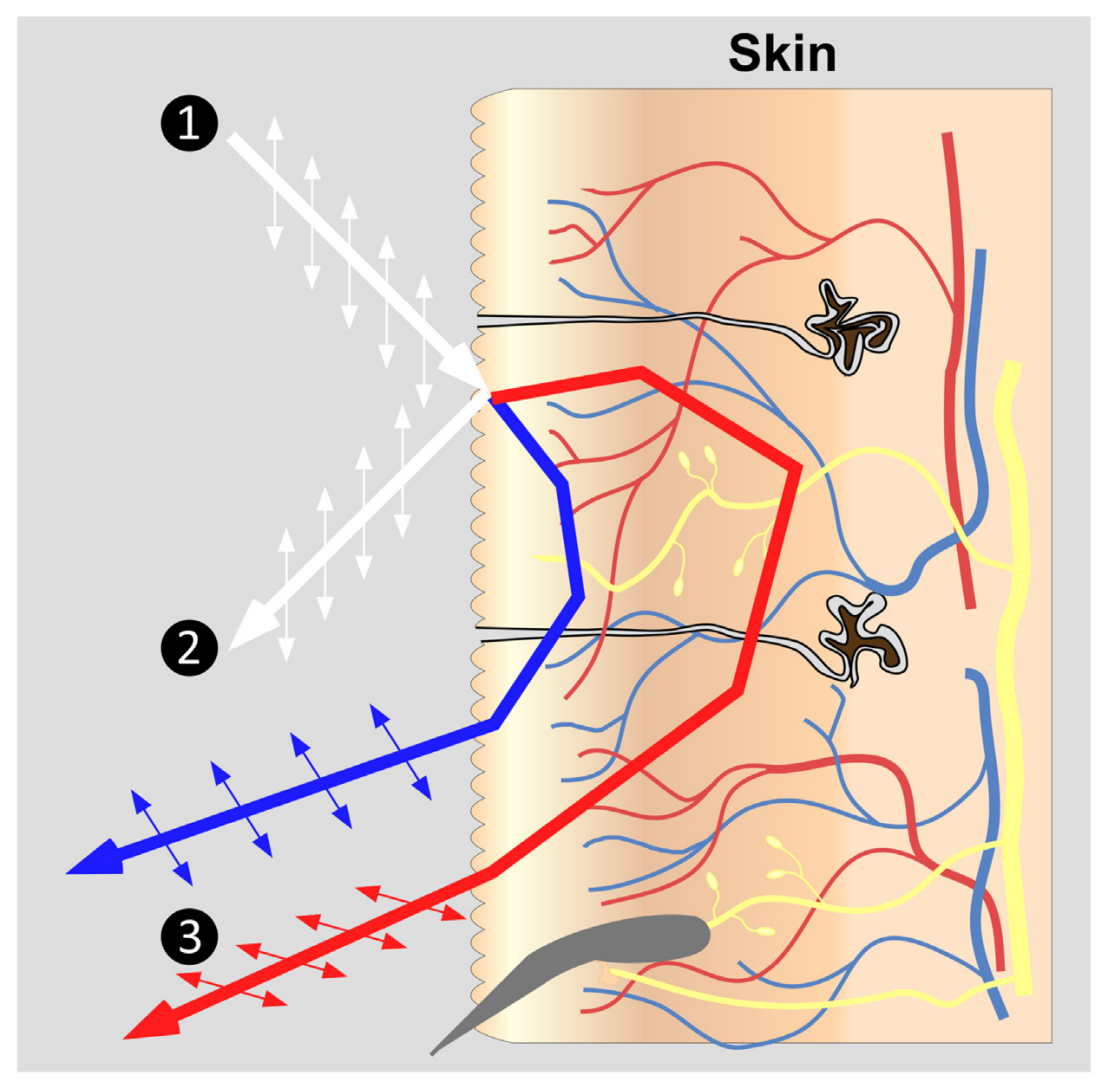
Light-tissue interaction.

**Illumination** The color and polarization of the illuminating light will contribute to the signal intensity and the level of surface detail of the fingerprint image. Figure 6 shows key characteristics of how light interacts with skin. In this example, we show polarized white light ❶ illuminating the skin. There will be a strong surface reflection with the *same* polarization as the incident light, shown by ❷. The remaining light will transmit into the skin where it will be absorbed and scattered and the polarization will be *randomized*. Some of the light will be scattered back towards the surface and exit back into the air for detection by the camera ❸. The blue component of the white light is highly absorbed and scattered by skin, such that any blue light that traverses deep into the skin will be absorbed and will never exit. Blue light that only interacts with near-surface skin layers can be scattered back out of the skin towards the camera. Red light, on the other hand, is less absorbed and scattered and it can travel farther into the skin. As a result, red light that returns out of the surface will have travelled deeper into the skin and can exit far away from where it entered, reducing the impact of surface features.

**Figure 7.**
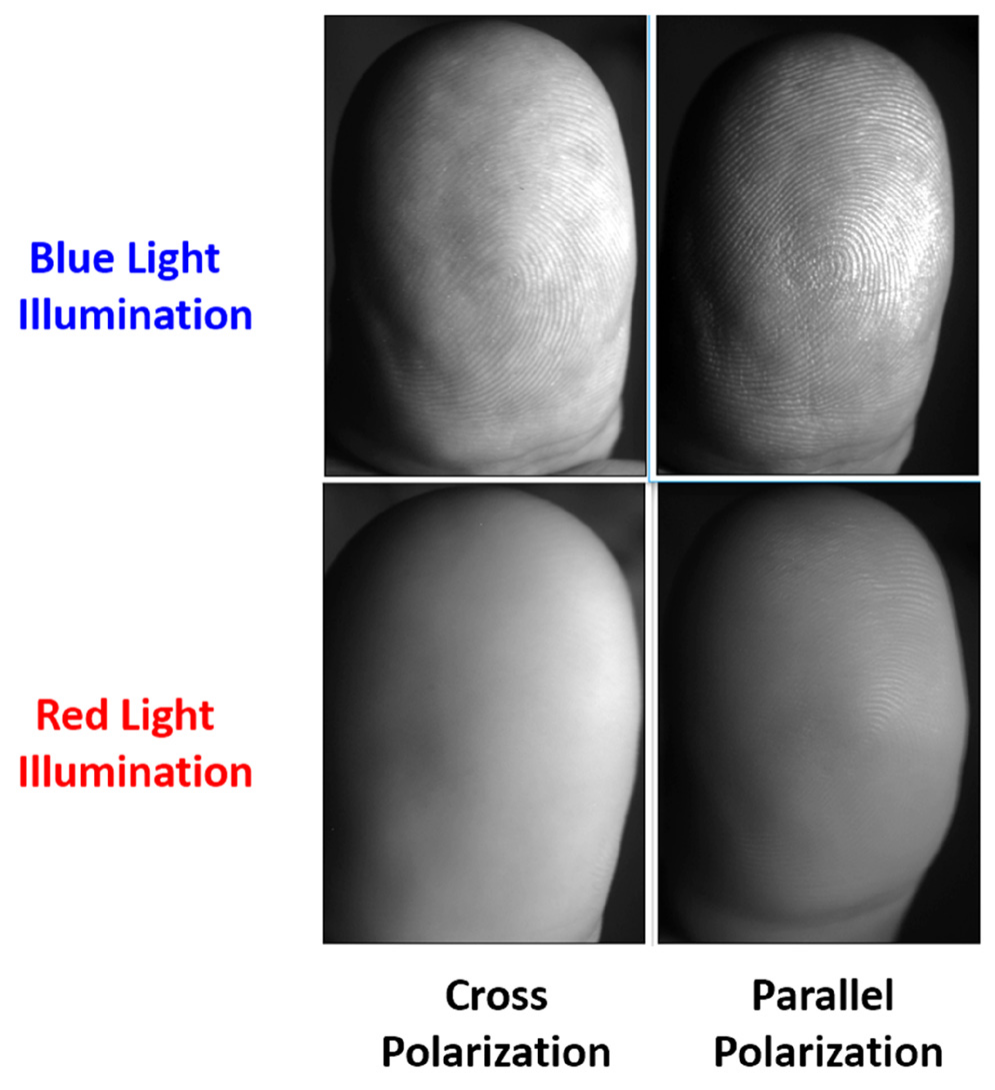
Effect of color and polarization on surface contrast.

Figure 7 shows how polarization and color changes impact the sharpness of the fingerprint image. Parallel polarization, where the illumination and detected polarization directions are the same, will create higher contrast images. Cross polarization between the illumination and detection will reject first surface reflections and primarily allow light that has scattered into the skin; thus subsurface features can be seen in cross polarized images ^18^. The effects of color and differences in penetration depth are clearly shown in Figure 7, with the blue light showing more surface features and the red light resulting in images with reduced contrast.

From this example, it may appear that a blue illumination with matched polarizer/analyzer is the best for surface contrast. It will produce the highest contrast images, but this also highlights another issue related to the intensity of the front surface reflections. When the illumination angle onto the skin is equal to the detection angle, high intensity specular reflections (i.e. glints) will occur and can saturate the camera. This effect is intensified when using a parallel polarization configuration. The top two images of the newborn in Figure 2 show a bright “halo” of light that is the result of specular reflections of the light source, which often results in camera saturation for those pixels. If the exposure time of the camera is set to reduce below saturation, there will be dimmer areas in the image that will have a poor signal-to-noise ratio (SNR). Since fingers are cylindrical, there is almost always a location on the finger that has this effect, depending upon the relative position of the light source. 

**Figure 8.**
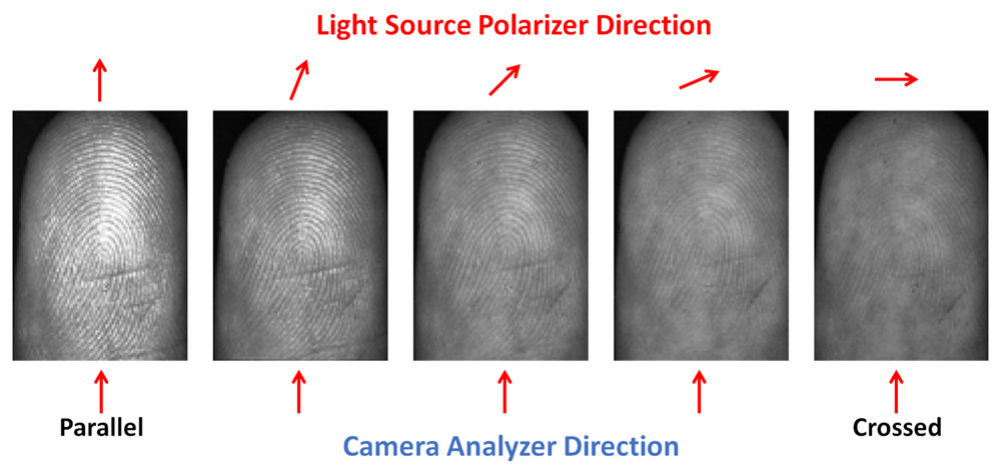
Effect of relative polarization angle on surface contrast.

This can be addressed in a number of ways. One is to conduct high dynamic range imaging, where multiple images are taken with different exposure times or illumination levels. Thus, a composite image can be created to reduce the bright spots due to specular reflections. For infants, however, this is very difficult since finger movement during multiple exposures is a near certainty and combining multiple images with movement would be computationally intensive and time consuming. Another method is to use other rotation angles between the illumination and camera. Figure 8 shows how surface contrast and specular reflections are affected by the relative polarization angle between light source and camera. Images start with parallel orientation on the left and the polarization angle of the light source is slowly changed until they are crossed on the right. Again, images show the reduction in both specular reflection and contrast as you move towards cross polarization. This highlights an issue with the parallel polarization, whereby images show hotpsots that require the camera to have a wide dynamic range. There is thus a potential to reduce glare, and reject surface features such as peeling, using appropirate combinations of color, polarization and illumination angle.

**Figure 9.**
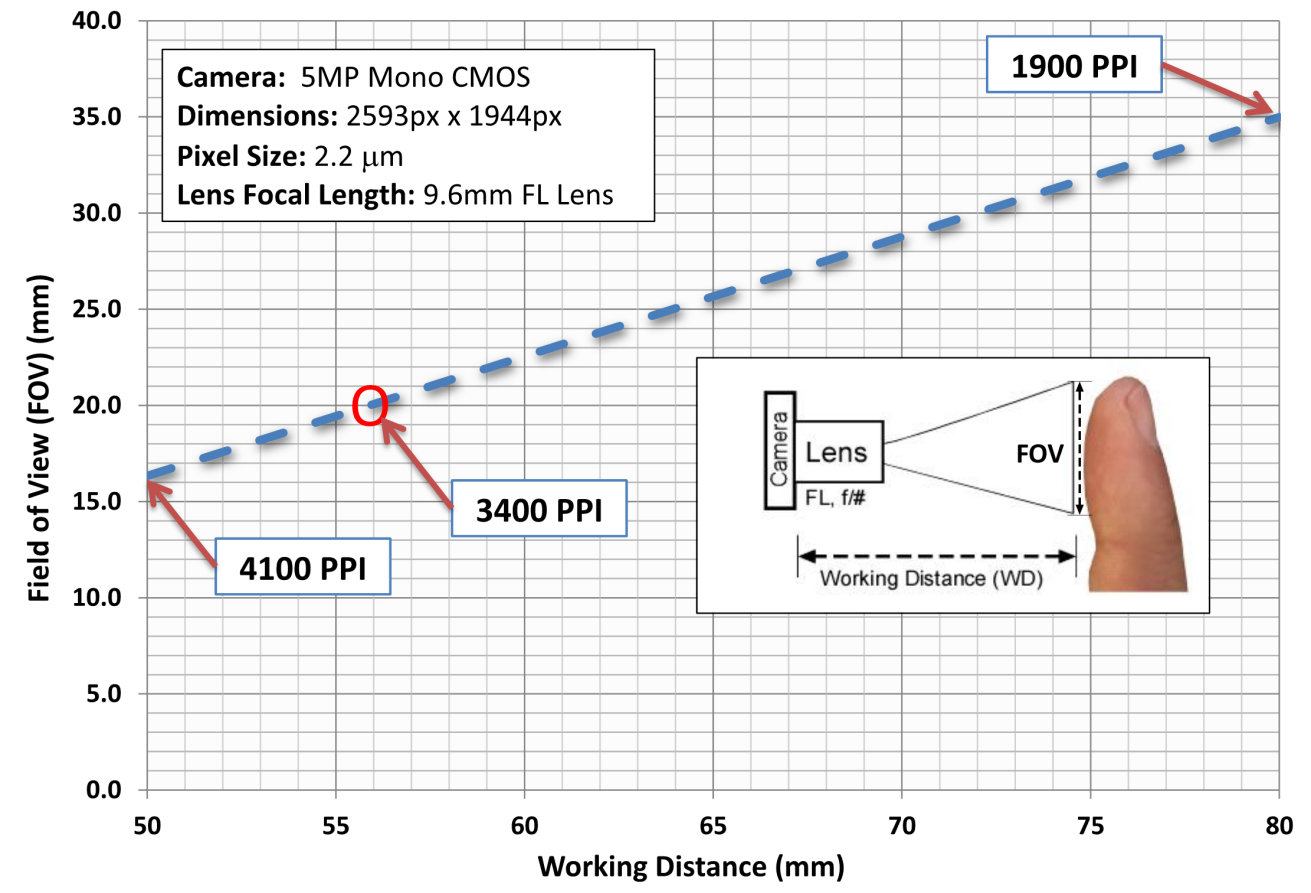
Trade-offs in field-of-view, working distance and resolution.

**Imaging system** Since the primary application for this device requires it to be small, low-cost and portable, we have relied primarily on commercial-off-the-shelf (COTS) components as much as possible. There is a standard class of board-level cameras that provide a small footprint and low power CMOS imagers in the 2–13-megapixel (MP) range with pixel pitches typically between ~1.5– 5μm. This class of camera is compatible with handheld devices using fixed focal length M12-style lenses with working distance of 2-3 inches. We selected a 5MP monochrome (b/w) camera with a 2.2 μm pixel size, coupled with a 9.6 mm FL lens (See3CAM_CU51, eCon Systems, India) We selected a b/w camera since it offers superior optical resolution and minimal aliasing with a single color (here blue) illumination. Additionally, we can pulse multiple colors during a burst of images in a manner similar to multispectral imaging (Lumidigm, USA), if desired. Figure 9 shows that with this camera/lens combination, we can achieve anywhere from 1900 PPI to 4100 PPI over reasonable working distances that accommodate a handheld device. Based on resolution needs discussed previously, we settled at 3400 PPI which gives a vertical image size or field-of-view (FOV) of ~20mm and provides ample resolution required for infants, but a large enough FOV to scan adults. 

**Figure 10.**
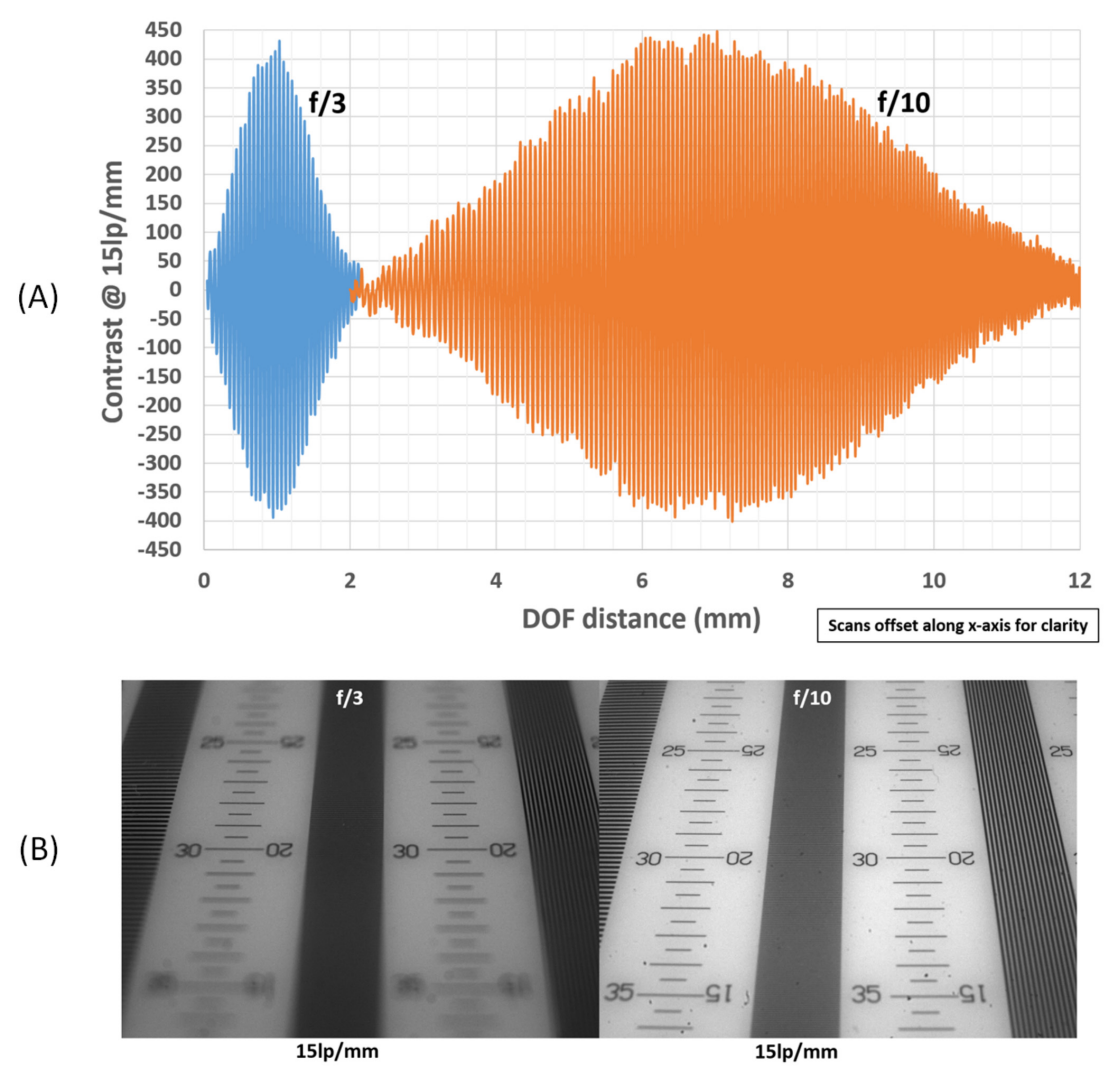
( **a**) Line scan showing contrast and depth of field at f/3 and f/10, and ( **b**) target images show improved depth of field at f/10.

An issue with fixed focal length M12 lenses is that they also have fixed apertures that lock it to a single, non-adjustable f/#, typically from f/2 to f/4, since these lenses are designed to work effectively in low-light surveillance applications. The lens we selected is fixed at f/3. The issue for our application is that an f/3 lens will have a short depth of field, especially at the WD that we are operating at. We can directly measure the depth of field using a 45-degree depth of field target that has scales consisting of horizontal lines at a frequency of 15-line pairs per mm (lp/mm), which is consistent with the feature sizes we are trying to resolve. Figure 10(a) shows the contrast for the 15 lp/mm scale as a function of distance, and we see that the contrast for the stock lens at f/3 goes to zero over a depth of field of 2 mm. This is a problem for non-contact fingerprinting, since the finger is not flattened and we need to stay in focus along the curvature of the finger. To improve the depth of field, we reduce the pupil of the lens with a custom laser-cut pinhole aperture to increase the f-number to f/10. Figure 10(a) shows the depth of field improves to 1 cm without reducing the contrast. Figure 10(b) shows the DOF target for these two configurations where the depth of field can be visualized, and the improvement is dramatic for the f/10.

A drawback of increasing from f/3 to f/10 is that the light throughput will be decreased by an order of magnitude. The lower light level is a concern because we are also trying to reduce any motion blur by keeping the exposure times less than 20 ms. To keep the motion blur to a minimum, we implement a custom LED light source to provide enough light to counteract the large f-number optics. These design goals would be difficult to achieve with a cellphone-based system, but with a dedicated purpose-designed device, we can make design decisions that make this possible. 

**Figure 11.**
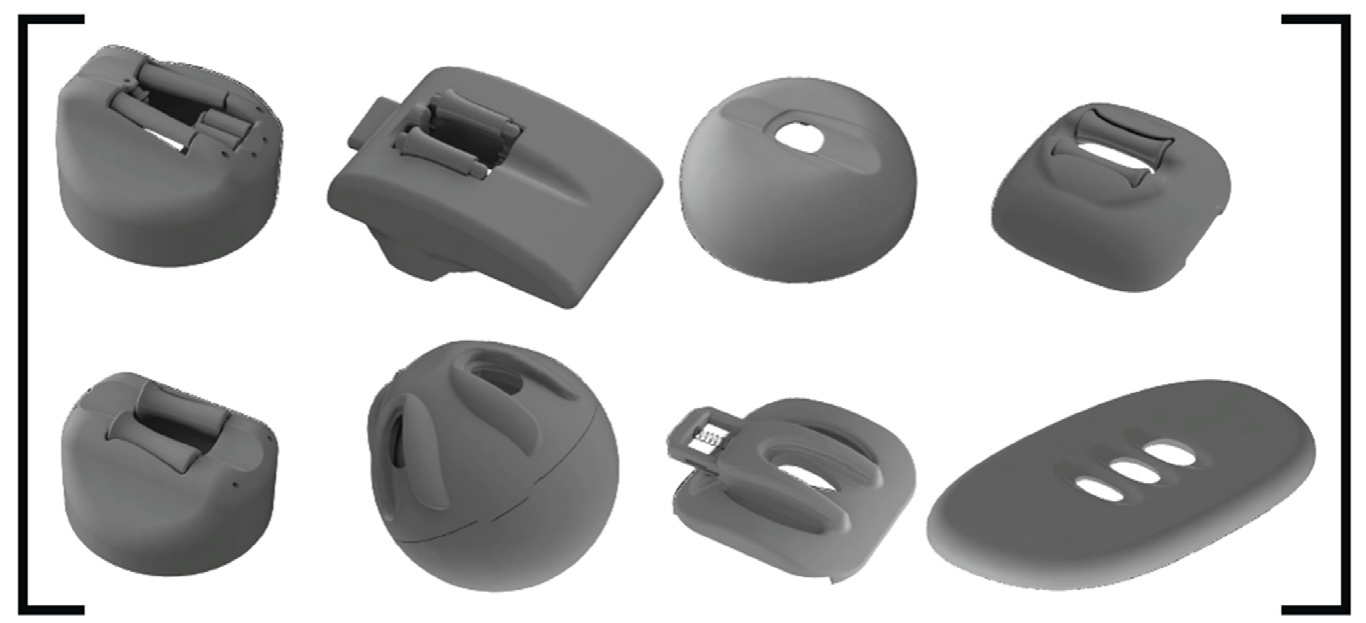
Examples of various non-contact hardware interaction designs (“tops”).

**Interaction design** Over the course of this effort, we developed and evaluated numerous experimental designs to understand what works best when the practitioner is interacting with the infant to properly place the finger. Figure 11 shows several such tops for the device to control placement of the finger. We have investigated a variety of design features, from adjustable apertures to rollers that permit rotation of the finger along its axis.

**Figure 12.**
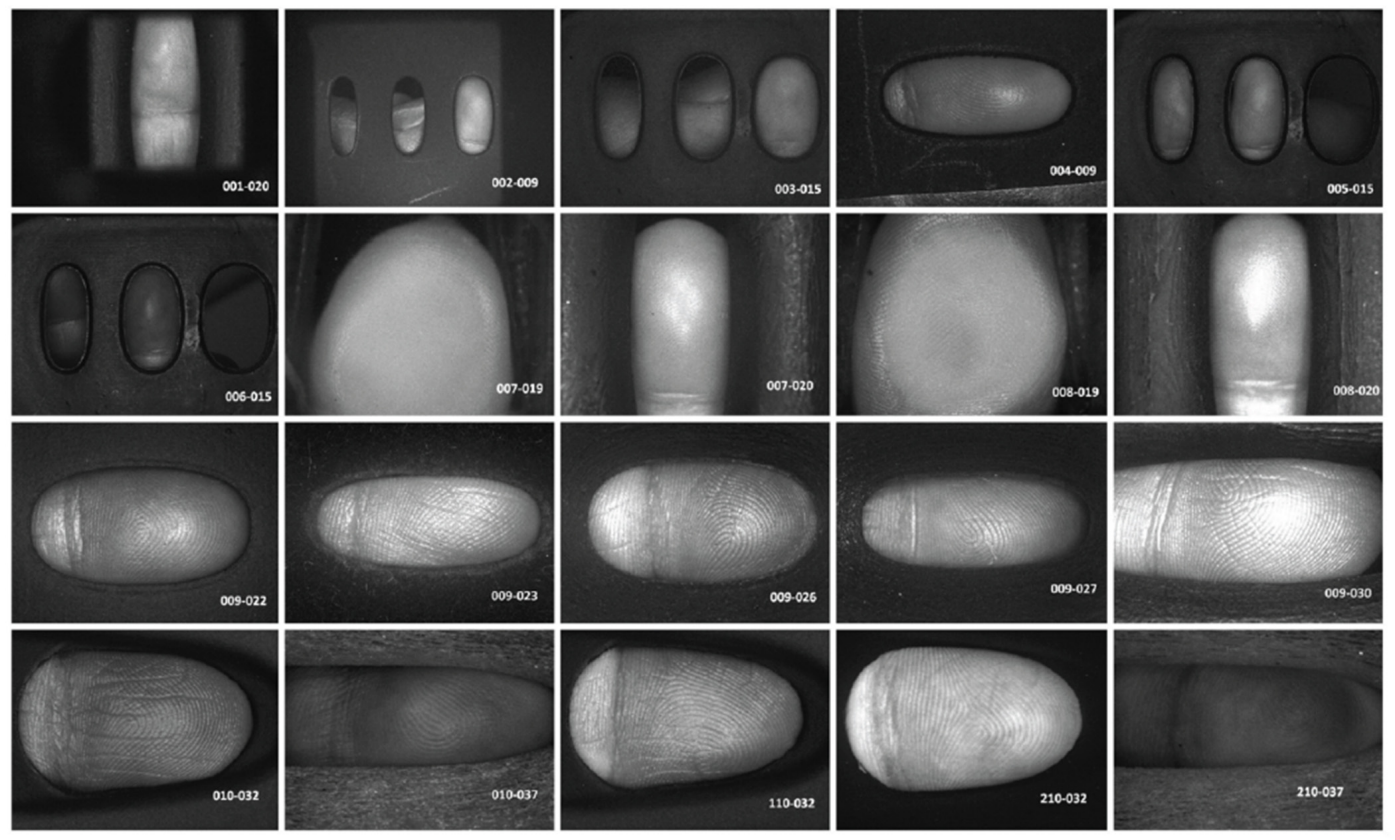
Imaging of fingers interacting with various top designs.

Figure 12 shows images collected for a variety of different tops, showing that we tested variations with different fields-of-view, resolutions, aperture shapes and sizes, contact/non-contact configurations, and a variety of illumination types (i.e. parallel/cross polarization, direct/diffuse light, etc.).

**Figure 13.**
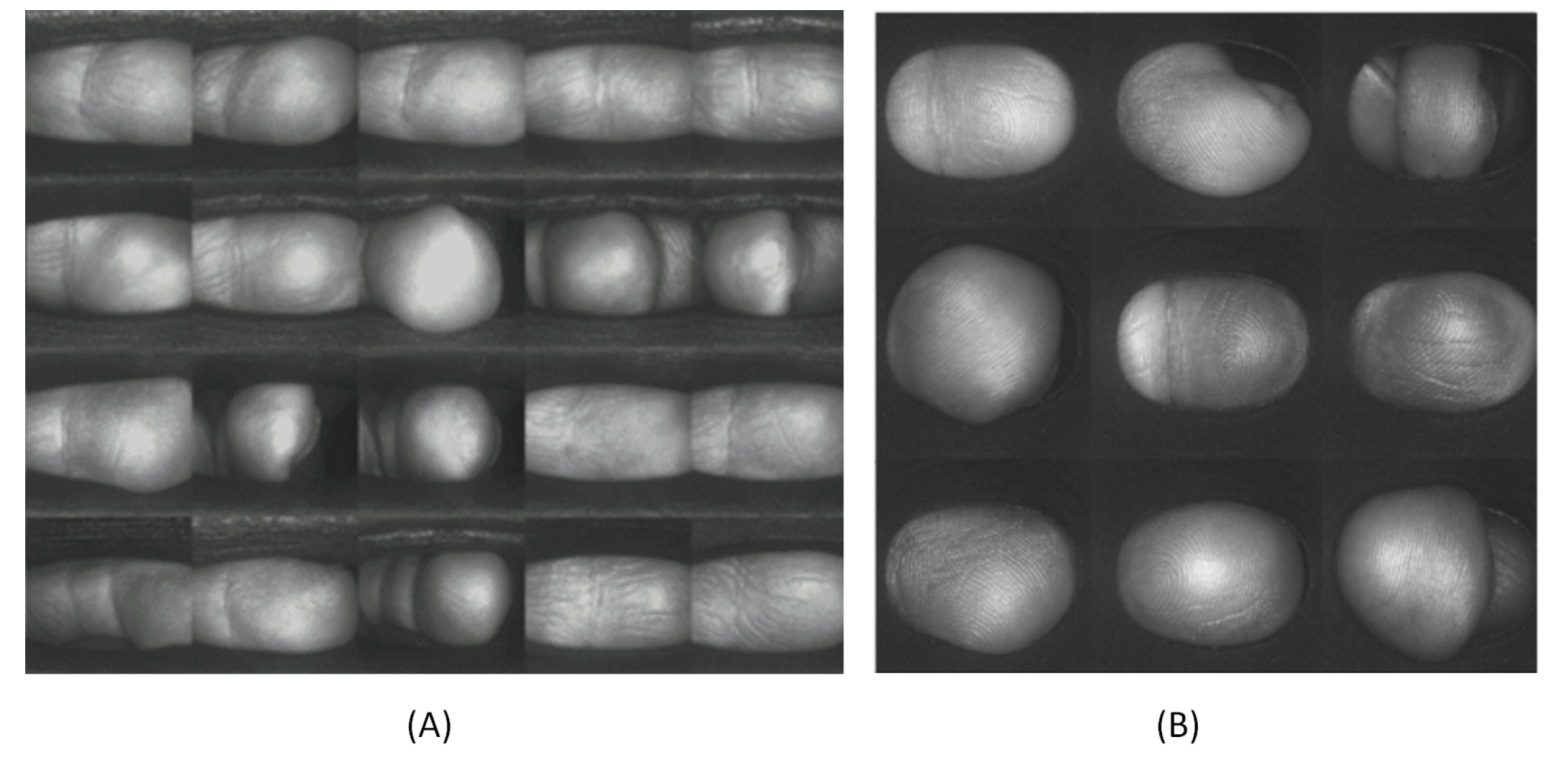
Examples of failure modes during finger placement. ( **a**) Images collected with a roller top, and ( **b**) images collected with a fixed aperture.

Our clinical testing shows how some of these designs failed, particularly due to the infant’s variable finger sizes and grasp reflex. Figure 13(a) shows a series of images that were collected with a roller-type top. We envisioned this configuration to be beneficial in that we could easily collect a series of images from nail-to-nail. The testing showed, however, that the finger placement is very inconsistent, and we could not reliably obtain quality images of the fingerprint. Figure 13(b) shows a similar issue with a static aperture. In this case, the size of the finger was too small for the aperture and it could easily poke thru the aperture during involuntary grasping. 

**Figure 14.**
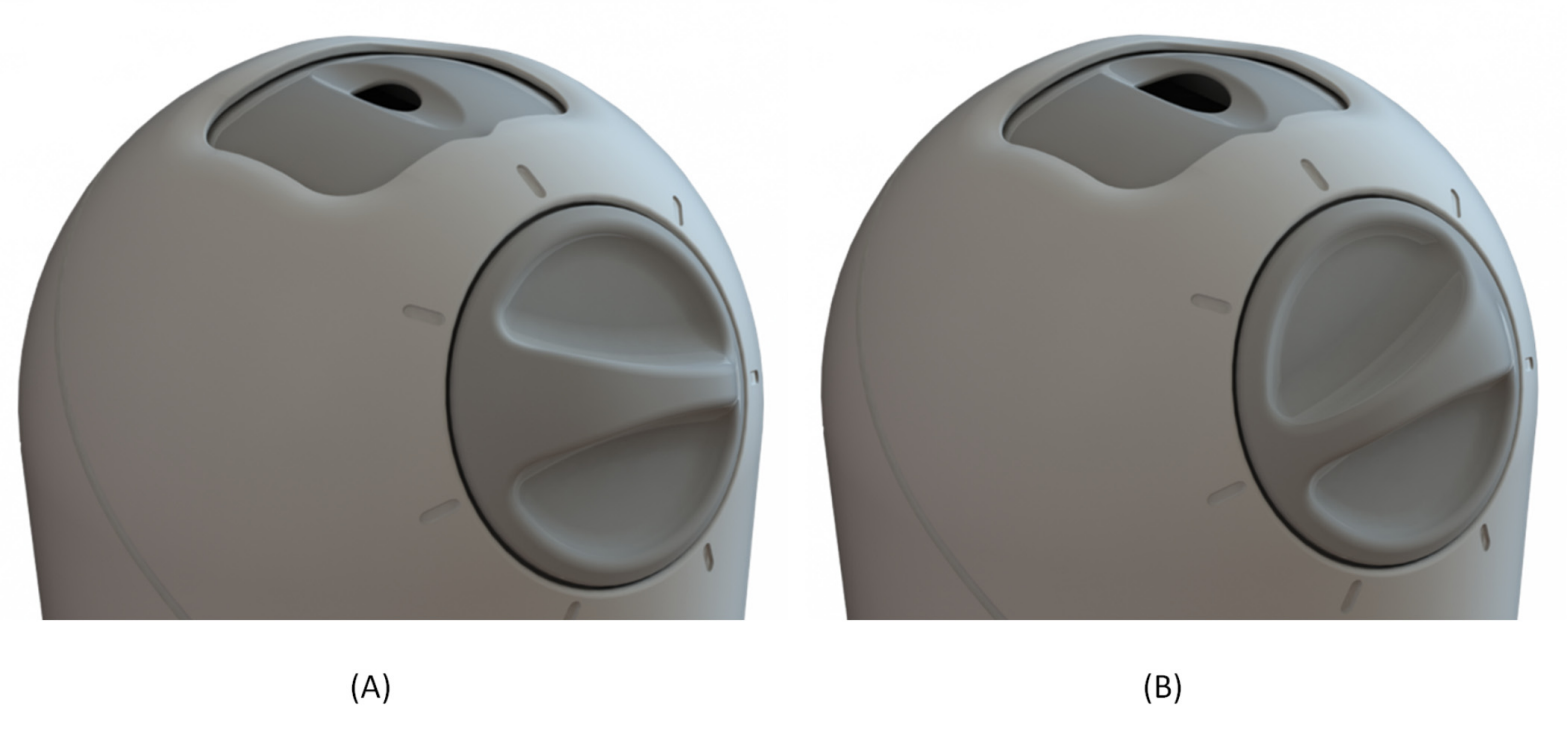
Optimized design with selectable apertures to accommodate size variations on a single hand or as children grow.

An infant’s finger size will vary over time, and there can be significant finger size differences for each hand, with a range of finger diameters from 5mm to 10mm. Some of the devices we developed had removable tops with different sizes, and others had adjustable or rotatable tops that could place apertures of different sizes into position. To accommodate fingers of different sizes and place them consistently, we developed an integrated aperture assembly that has multiple positions, as shown in Figure 14.

**Figure 15.**
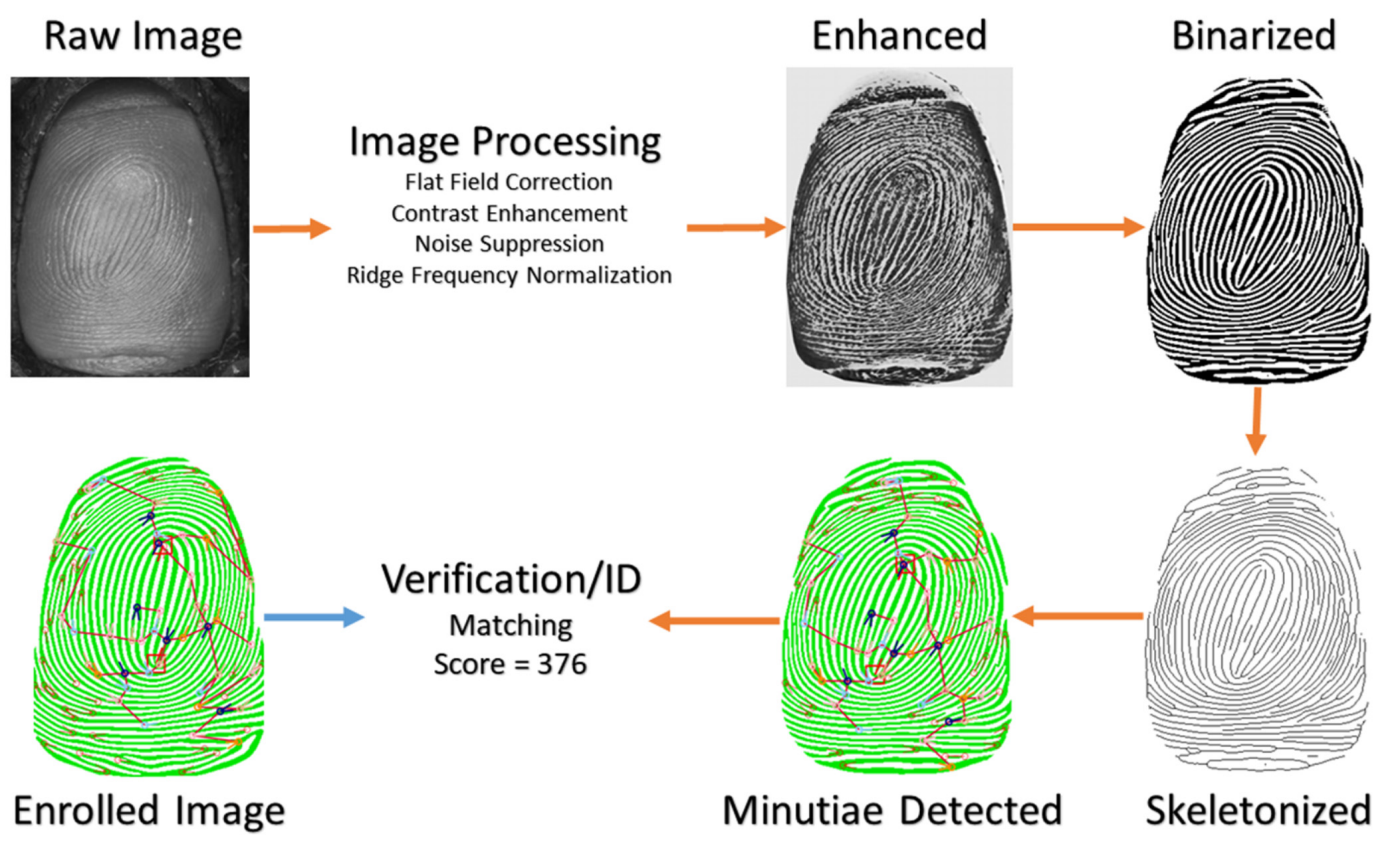
Image processing pipeline.

**Image processing** A distinct advantage of ink on paper or FTIR-based contact fingerprints is the high contrast images that are created. Other than resizing, very little image processing is required prior to minutiae detection. For a non-contact imaging device such as ours, however, the print ridges and valleys do not have the same high contrast upon collection and the finger curvature can present issues with uneven illumination. Figure 15 shows our image processing pipeline with custom image processing to accept the monochromatic 12-bit raw image, flat-field correction to eliminate lighting non-uniformity, contrast enhancement, noise suppression and ridge frequency normalization. The manipulated/enhanced image is then resampled to a reduced pixel count with an 8-bit pixel intensity range. The resultant enhanced image is then evaluated with commercial software (e.g. Megamatcher, Neurotechnology Inc., Lithuania) to create the binarized and skeletonized images, followed by standard, interoperable, template creation and minutiae detection which can then be used for verification and/or identification to stored templates, allowing interoperability between systems ^19^. This is a distinct advantage over “black-box”, machine learning approaches that suffer from lack of interoperability and legal acceptance.

**Figure 16.**
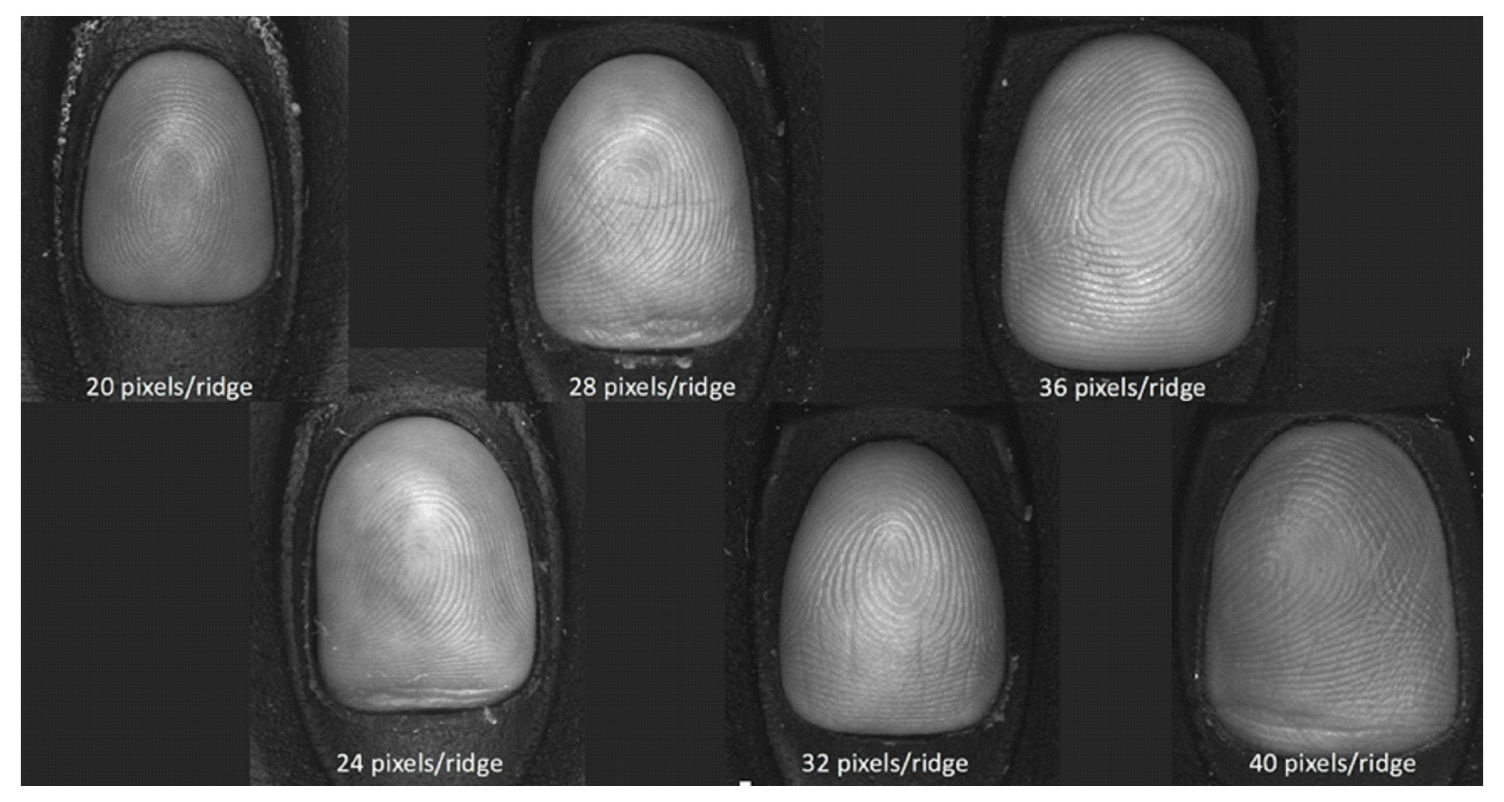
Variations in infant fingerprint ridge density.

The ridge frequency normalization step is implemented because we are using commercial software that was developed to evaluate standard adult contact fingerprints. As discussed previously, adults have a ridge-to-ridge distance of approximately 450 microns and that dimension is relatively consistent across adults. In addition to being much smaller than adults, infants and children are growing rapidly and their finger sizes will have much larger variations across individuals and for any infant over time. Figure 16 shows examples of infant fingerprints using our system; the spacing of the ridges, measured in camera pixels, is shown to vary by at least 2x from 20 pixels/ridge to 40 pixels/ridge, whereas adults will be relatively stable at about 60 pixels/ridge. 

**Figure 17.**
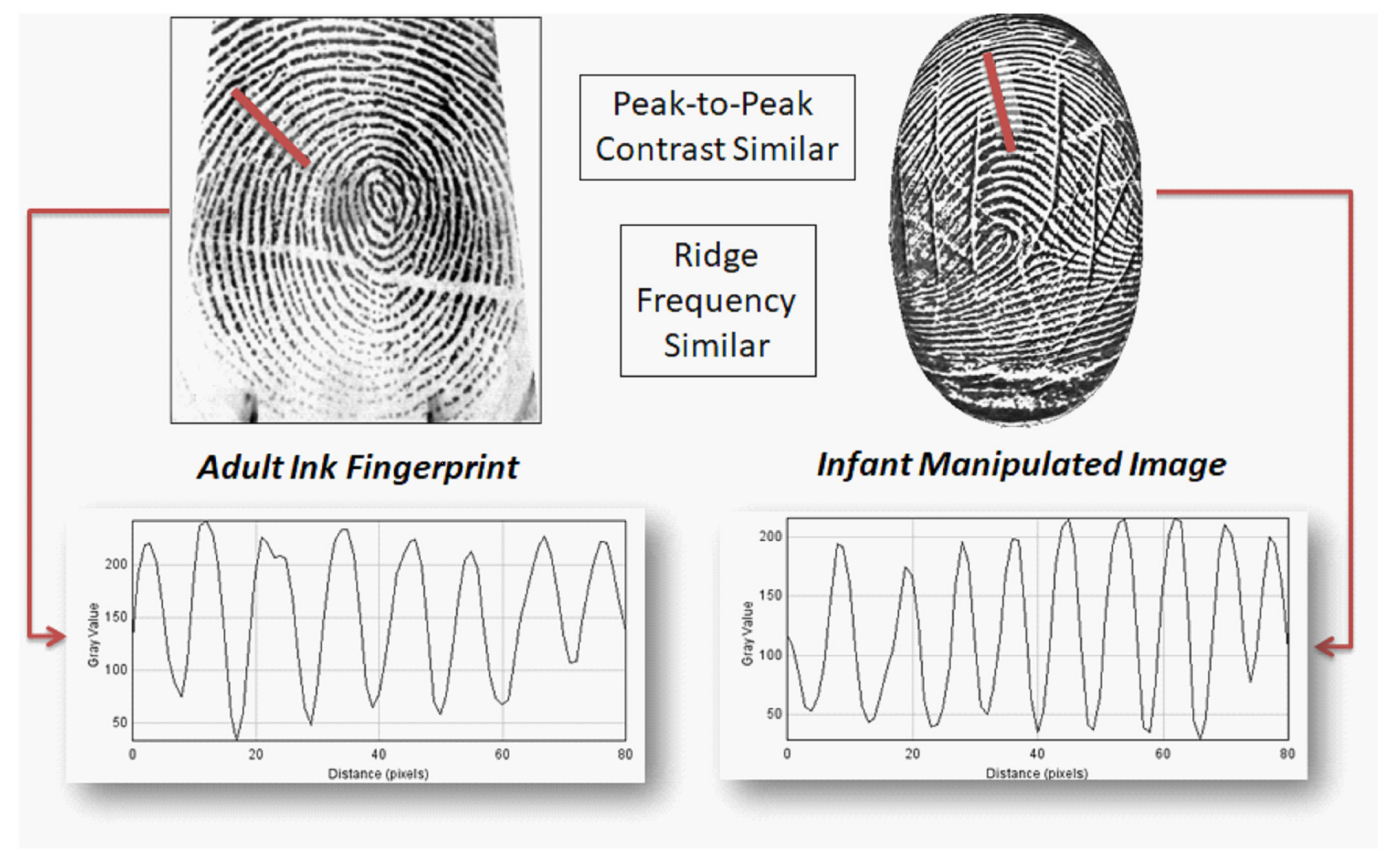
Appropriately acquired and processed infant fingerprints have similar characteristics to adult prints.

**Figure 18.**
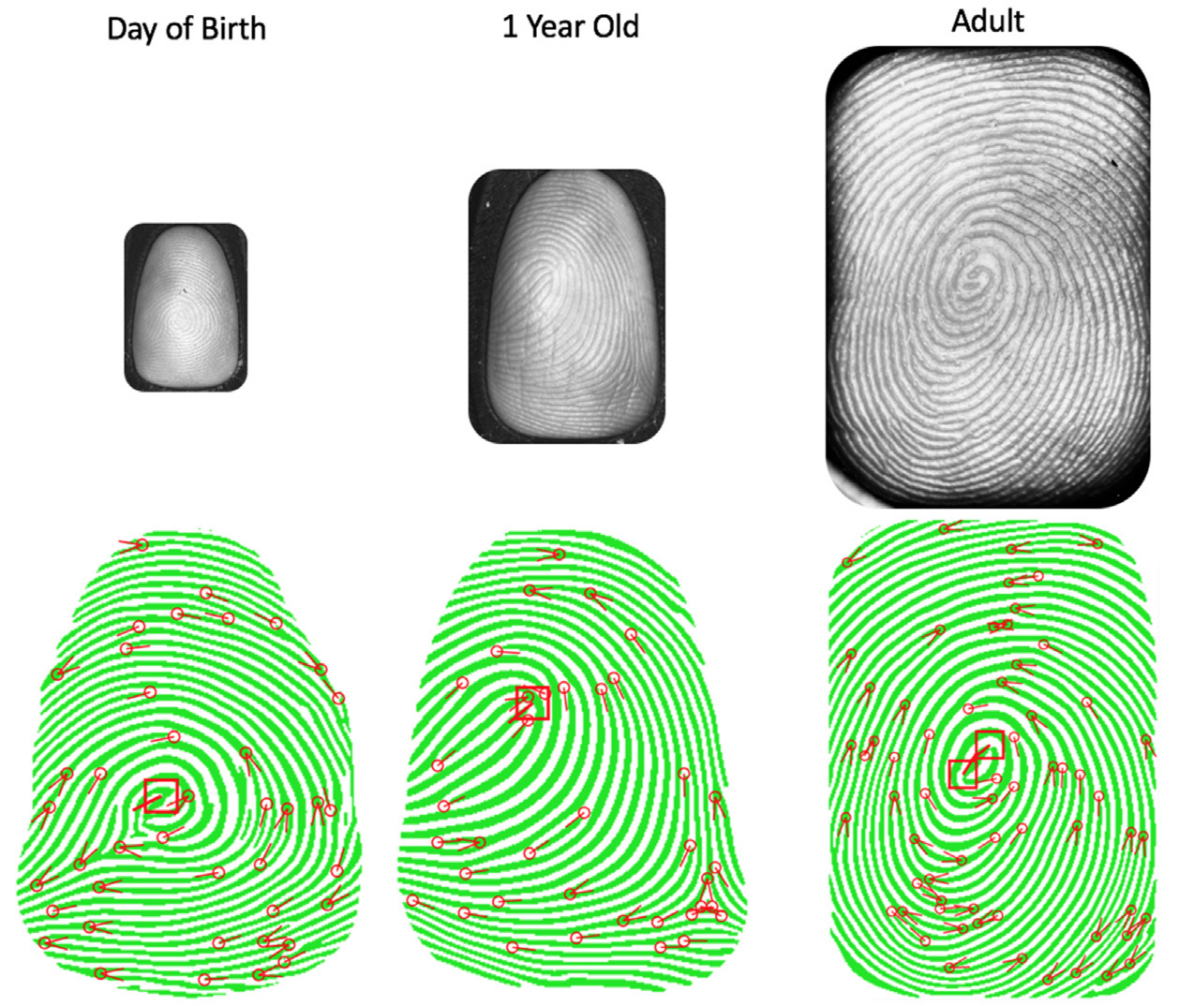
Newborn, 1-year old, and adult images (top) with processed templates and minutiae (bottom).

Within our image processing pipeline, the frequency of the fingerprint in each raw image is calculated and that value is used to resample the image such that the ridge distance is corrected to be 8 pixels prior to input into the fingerprint analysis algorithms. Figure 17 shows a standard ink print of an adult on the left, and a resampled, frequency corrected image of an infant on the right. The plots show the light intensity across the ridges of the print at the red line and it is seen that in both the adult and infant print the pixel density is ~8 pixels from ridge-to-ridge. This pixel density is what the image processing algorithms use to create the binary and skeletonized images expect. If the pixel density is too high, as in our high-resolution raw imagery, the binary reconstruction and minutiae detection algorithms will often fail. Figure 18 shows fingerprint images taken with our device for a child within 12 hours of birth, a one year old, and an adult. The ridge-to-ridge frequency in the raw imagery pixels/ridge will change from 20 pixels/ridge in the newborn to 60 pixels/ridge in the adult. By resampling to ~8 pixel/ridge, we can effectively remove any issues related to size and age. As the bottom row of Figure 18 shows, the binary images created can all be readily analyzed by the minutiae detection algorithms without size/age being a limiting factor. 

### Clinical performance

Over the course of this effort, we tested many designs, varying cameras, lenses, light source configurations, finger holding methods, and algorithm variations. Lab analysis is instructive and testing on an adult or infant in a research environment is good, but we have learned that these situations are not representative of the infant population, nor do they accurately predict the caregiver/infant interaction. We determined that the only true way to quantify whether a design is effective is to use the device in a real-world environment and evaluate the quality of the images as well as the ability to match fingers, both in single sessions and longitudinally over time.

We recently completed our first international clinical testing effort of 500 newborns and infants <6 months of age at Tijuana General Hospital, Mexico, with multiple follow-up visits possible over 12 months as infants return to the hospital for vaccinations or baby check-ups. This study was carried out with informed parental consent and approved jointly by UC San Diego IRB and TGH ethical committees (UCSD IRB#151400). The effort was structured as a two-phase pilot, with Phase I designed to evaluate numerous hardware, software, and protocol variations for system performance optimization. This phase utilized US biometricians with local nursing support. For Phase II, the system design was locked with local staff operating the system and collecting images. The goal for Phase II was to provide performance data with consistent hardware/software configuration and to evaluate system usability with local users.

**Figure 19.**
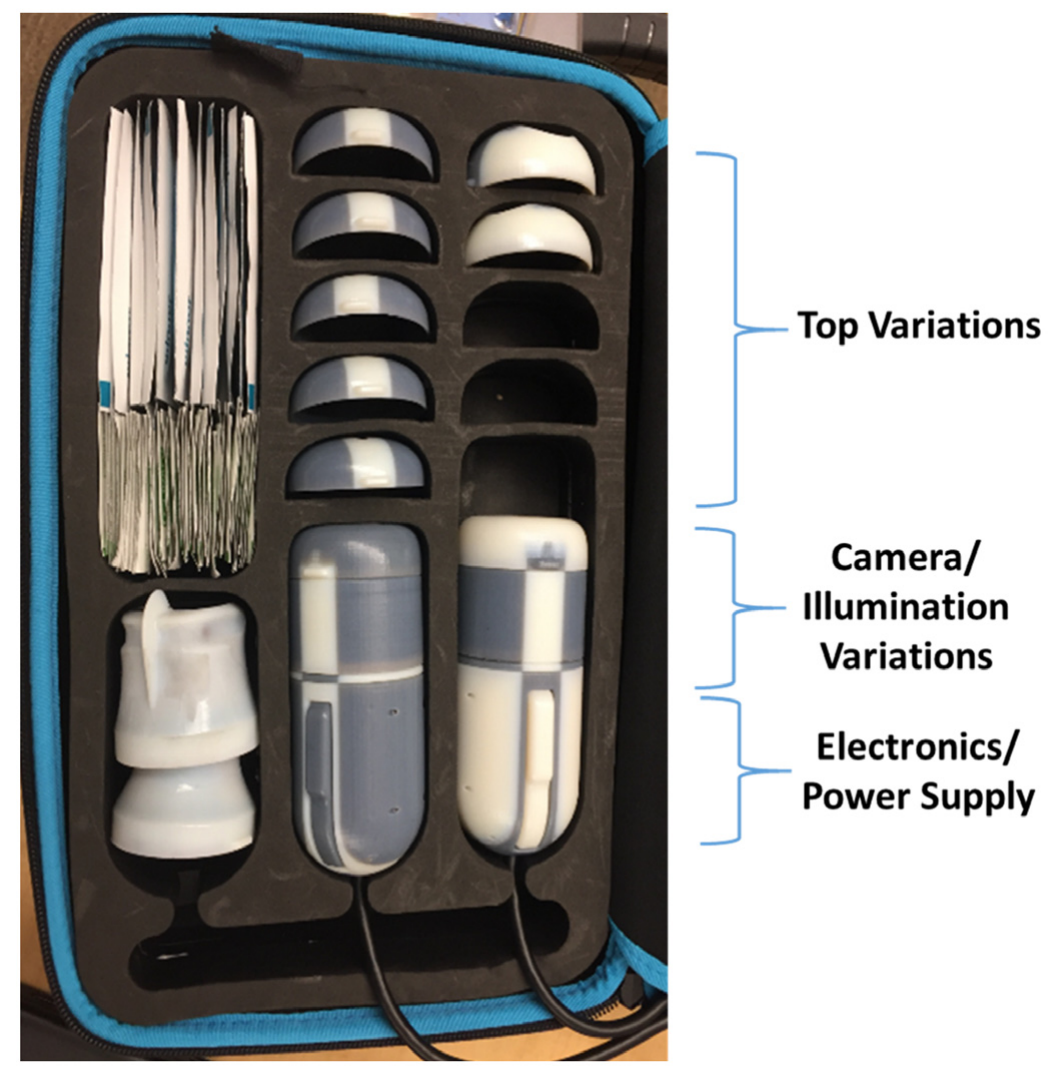
Phase I field-kit.

**Figure 20.**
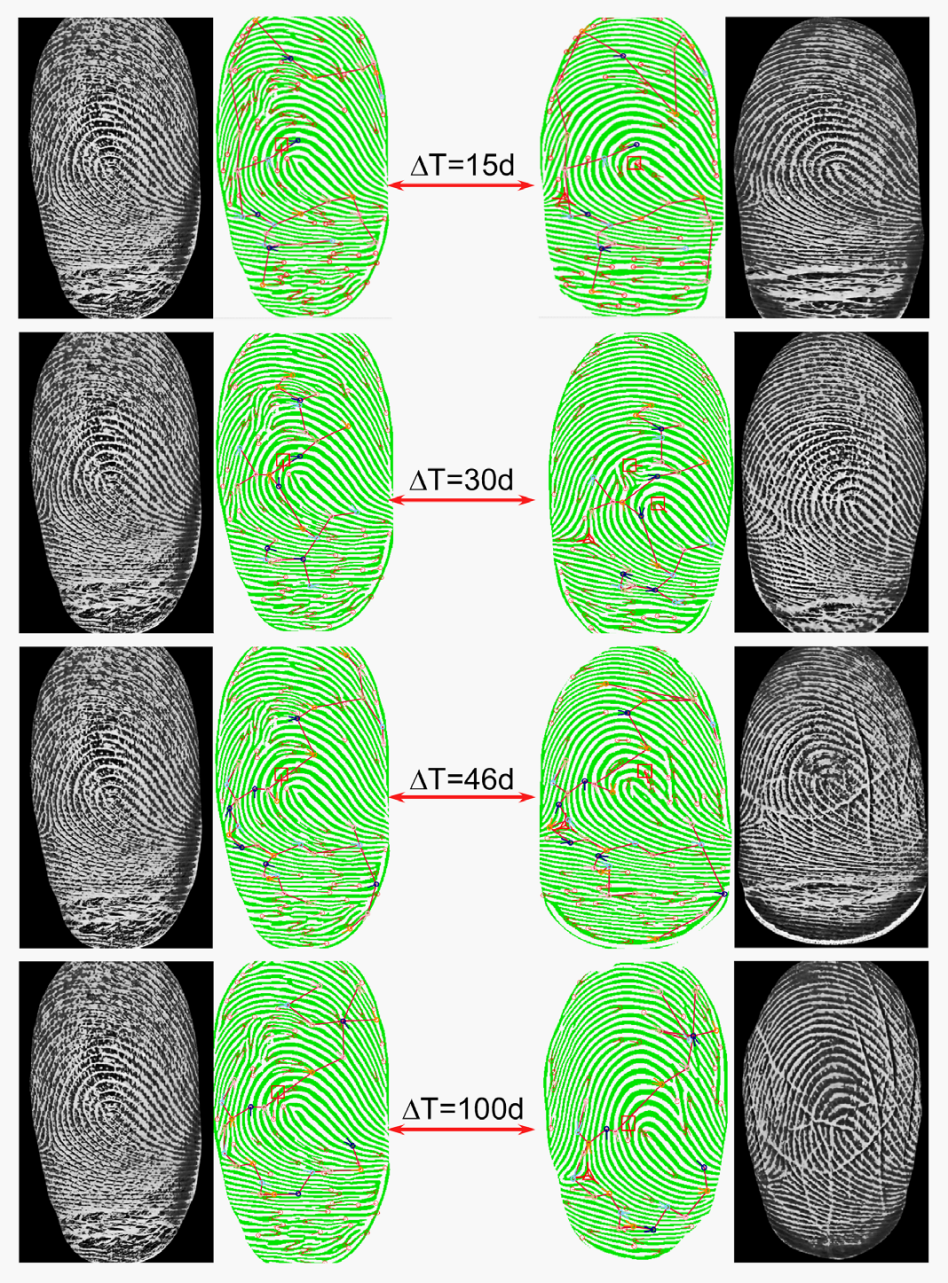
Persistent matching of a single infant over multiple visits.

The initial technology assessment from the early Phase I study utilized a modular construction so that we could modify any subsystem for testing. Figure 19 shows the clinical kit with multiple devices configured in a variety of ways. The modular nature allowed us to vary any of the subsystems, providing the ability to test different configurations on the same infant during any session. Figure 20 shows an example of a single subject enrolled at 26 days (left column) and verified four times over the next 100 days (right column). The green binarized images show minutiae maps indicating correct identification of the infant at each of the visits.

**Figure 21.**
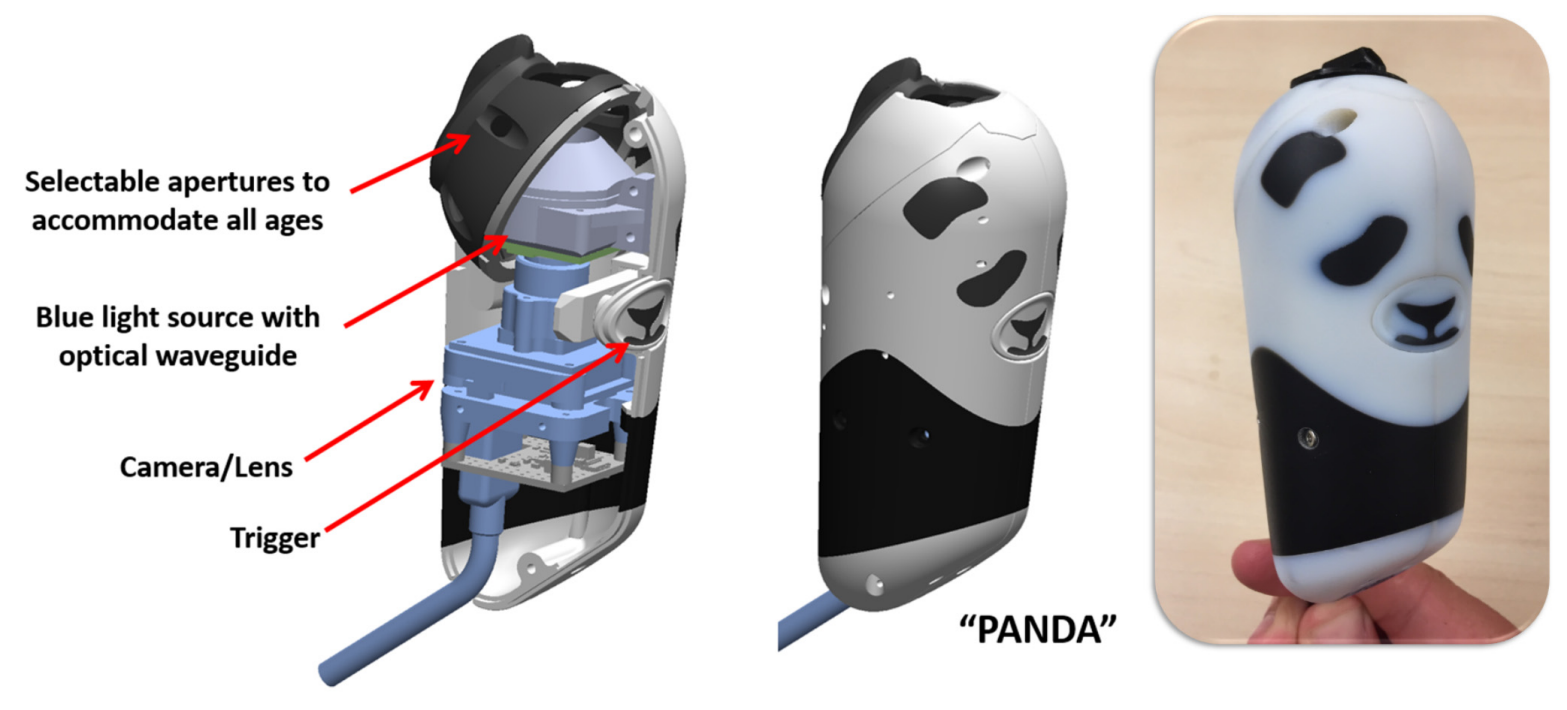
Device deployed for clinical testing.

As we moved to Phase II of the effort, we established a more stable design better suited for deployment in the field with local users. As a result, we stabilized the design to provide consistency over time, while retaining device adjustability when required. Figure 21 shows the device in a kid-friendly “Panda” design. This optimized configuration currently delivers blue LED lighting through a concentric wave guide that projects dispersed light at an optimal angle on the subject, while rejecting both specular light from the finger and stray light to the imager. Images are collected at high resolution by a monochrome CMOS imager with custom optics to optimize resolution at 3400 ppi, a depth of field > 1 cm, and exposure times of < 20ms to minimize motion artifacts.

Preliminary results from the first 100 paired visits from this study are very encouraging, with 100% re-identification- even when enrolled on the day of birth. Results of the full trial, including comparisons of non-contact finger, palm-pad and palm printing, will be published in subsequent articles.

### Preliminary results

In order to validate and optimize image processing performance prior to analyzing the full clinical trial dataset (504 kids, 44,953 fingers, median time between enrollment and verification ΔT = 83 days, max ΔT = 388 days), we performed detection error tradeoff (DET) analysis on a random subset of these data (1815 single finger enrollment/verification true pairs and 4808 false pairs from 142 infants). We varied key parameters, including blur radius, bimodal stretch noise floor, and ridge-to-ridge distance correction, maximizing average matching score for true positives and minimizing average matching scores for false pairs. This analysis yielded values of 24 pixels for smoothing, and 64 DN’s for bimodal stretching, at a resampling of 8 pixels/ridge-to-ridge distance from the 3400 PPI acquisition resolution to standard 500 PPI template creation.

**Figure 22.**
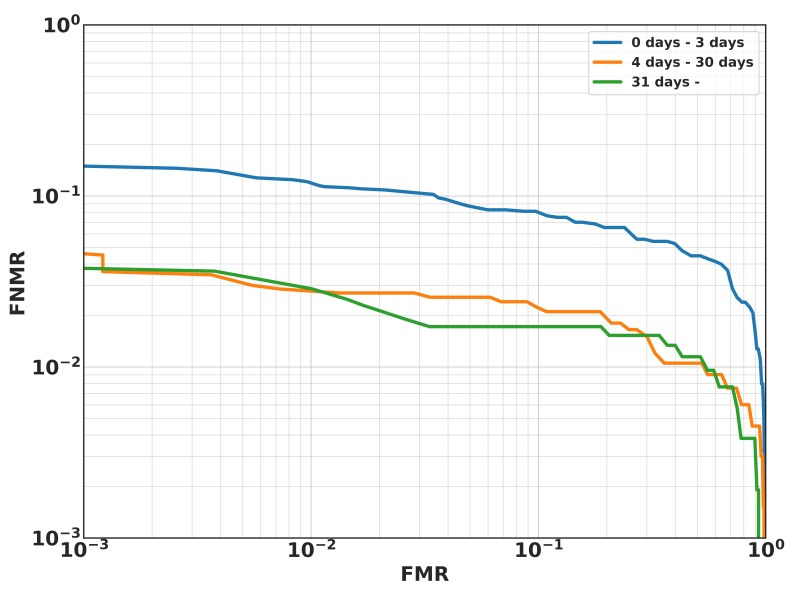
Preliminary Detection Error Tradeoff Analysis.

DET curves generated with these parameters are shown in Figure 22. Data are categorically separated by age at enrollment: newborn = 0–3 days (n=48); neonate = 4–30 days (n=58); and infants > 30 days (n=98). For newborns, we find per finger TAR = 85.0%; for neonates = 95.4%; and for infants = 96.2%, at FAR = 0.1%. The verification rate for these 142 individuals was 99.4% for newborns and 100% for age > 3 days, when more than three fingers were enrolled.

Results of the full trial analysis, including comparisons of non-contact finger, palm pad and palm printing, will be published in subsequent articles.

### Summary of lessons learned

Broadly, there is a gap in reliable biometrics for infants and children, and we find that there are a few critical considerations in designing biometrics newborns and neonates in particular ( Table 1). The first is the realization that technology itself can be a failure mode and thus human-centered methods that assure the right problem is being solved and couple design with rapid and agile prototyping in real world settings are often needed. From study of the literature, and our own experience, we propose that fingerprints remain the most promising biometric because they are established before birth, remain persistent throughout life, are acceptable to caregivers, and can be acquired and shared in interoperable formats. Other biometrics such as iris-scanning are technically possible but have multiple and significant failure modes when acquired in the field. Likewise, machine learning approaches can be applied to any of these biometrics, yet these methods lose explainability and interoperability with accepted techniques. Finally, the design of any infant fingerprinting technology must account for behaviors, physiology, ethical and social concerns unique to this population.

**Table 1.** Summary of Lessons Learned.

• Use Human-Centered Design methods over technology-centric development approaches.
• Test early, iterate often, improve design.
• Performance cannot be measured by lab tests and/or small number of infants. Longitudinal testing, with many infants, across multiple ages, in realistic environments is required.
• Fingerprints as a primary infant biometric have potential advantages of uniqueness, acceptability, persistence, and interoperability of templates.
• Other biometric identification modes besides fingers, especially iris scanning, are technically possible but fail due to infant behaviors (e.g. closed eyes), confounders (e.g. eye infection, trauma or irritation), or social factors (e.g. certain parents/certain cultures do not tolerate scanning of infant faces). Ears remain a promising secondary biometric in many settings and may improve over time.
• Infants are “uncooperative” and have delicate physiology--so technology needs to work with infants--not the other way around.
• Newborn skin peels in the first weeks of life, and this sometimes requires scanning of multiple fingers or palms to find quality regions.
• Contact sensors are problematic due to the softness/deformability of infant fingers.
• Standard 500 PPI resolution is not adequate to scan infant fingers. Increasing to a resolution of 3400 PPI helps capture small features in non-contact mode.
• Use blue light and not white light. The red portion of white light will penetrate too far into skin and will cause surface features to be less pronounced.
• Use a b/w camera with exposure times <20 ms to optimize resolution and reduce motion blur.
• Use polarized illumination and polarized detection to enhance surface features and reject light that has scattered deeper into the skin.
• Significant image processing and ridge frequency normalization is required to conduct non-contact biometric analysis.
• Positioning is critical to matching performance, and hardware design and/or interaction protocol must deal with this.
• Size/shape of the finger aperture impacts accuracy. Designs should help align the finger in a manner that supports consistent imaging.
• Based on the above, a dedicated device that facilitates proper interaction, controls illumination, depth-of-field, field-of-view, and resolution, is needed to ensure consistent biometric capture.

## Conclusion

We used human-centered design to reframe the infant identity problem and develop new methods for biometric capture. We worked with children from birth through 24 months, along with parents, caregivers, nurses, doctors, health officials, and vaccinators, to build technologies that accommodate human physiology and behaviors rather than trying to control them. We tested prototypes of increasing sophistication in an iterative process with infants in real-world conditions. As a result, we accumulated numerous lessons learned, resulting in optimized procedures and a system design that allows effective fingerprinting of infants, children and adults.

## Data availability

### Underlying data

**Replication Data for Biometric Identification of Newborns and Infants by Non-Contact Fingerprinting: Lessons Learned. ", https://doi.org/10.7910/DVN/PSQHNJ^20^**

These files contain data corresponding to all published figures in this article.

All data underlying the results are available as part of the article and no additional source data are required
