# Supplementary material for: Biometric recognition of newborns and infants by non-contact fingerprinting: lessons learned
Source: Gates Open Res. 2019 Nov 5;3:1477. Originally published 2019 May 29. [Version 2] doi: 10.12688/gatesopenres.12914.2 (PMC6667827; doi:10.12688/gatesopenres.12914.2)
Supplement: Peer review history

# Peer review: Reviewer response for version 2

**DOI:** 10.21956/gatesopenres.14218.r28179

**Published:** 2019-11-18

**Authors:** Sunpreet S. Arora

**Affiliations:** 1 Visa Research, Palo Alto, CA, USA

I believe they have addressed most of my major concerns and acknowledged that they are working toward addressing the rest in a follow-up manuscript.

I confirm that I have read this submission and believe that I have an appropriate level of expertise to confirm that it is of an acceptable scientific standard.

# Peer review: Reviewer response for version 1

**DOI:** 10.21956/gatesopenres.14013.r27439

**Published:** 2019-07-30

**Authors:** Silvio Barra

**Affiliations:** 1 Department of Mathematics and Computer Science, University of Cagliari, Cagliari, Italy

I think that the proposed paper is really well written. Recognition of infants by means of contactless biometric can save them from a plenty of issues, like cradle swapping and related. 

 The contactless fingerprint recognition is a very captivating argument to focus on when speaking about babies recognition, and I think that the times are perfectly mature to approach the topic. In this work, a very informative and interesting analysis is done over this argument, as well as an interesting analysis is done.

 The only suggestion (it is more a feedback) is about the use of the words identification, recognition (1:N matching) and verification(1:1 matching).

 I've seen that a "babies biometrics related works" section is missing, and I suppose that this could be very interesting and also it could complete a very good paper. I attach here three potential papers which deal respectively with ballprint, ear and face recognition on infants. These can fit this section if you aim at inserting it.

I confirm that I have read this submission and believe that I have an appropriate level of expertise to confirm that it is of an acceptable scientific standard.

## References

[1] Ballprints as an infant biometric: A first approach. *2014 IEEE Workshop on Biometric Measurements and Systems for Security and Medical Applications (BIOMS) Proceedings*.2014;36-43

[2] Biometric authentication of newborn identities by means of ear signatures. *2014 IEEE Workshop on Biometric Measurements and Systems for Security and Medical Applications (BIOMS) Proceedings*.2014;1-7

[3] : Intelligent Method for Face Recognition of Infant. *International Journal of Computer Applications*.2012;52(4) : 10.5120/8194-1569 36-50 10.5120/8194-1569

# Response

**Published:** 2019-10-08

**Authors:** Eliah Aronoff-Spencer

Thank you to reviewer three for your comments and suggestions. See below for responses as well as updates to the paper.. 

1. “The only suggestion (it is more a feedback) is about the use of the words identification, recognition (1:N matching) and verification(1:1 matching).”

- We appreciate the feedback and have made updates to the paper. 

2. “I've seen that a "babies biometrics related works" section is missing, and I suppose that this could be very interesting and also it could complete a very good paper. I attach here three potential papers which deal respectively with ballprint, ear and face recognition on infants. These can fit this section if you aim at inserting it.”

- We appreciate the comment. Note that references 3-10 review the current state of infant biometrics including ballprints, ear and face recognition. Likewise cited works by Jain *et al.* give thorough reviews of historic works.

# Peer review: Reviewer response for version 1

**DOI:** 10.21956/gatesopenres.14013.r27436

**Published:** 2019-07-17

**Authors:** Sunpreet S. Arora

**Affiliations:** 1 Visa Research, Palo Alto, CA, USA

The authors present a method for biometric recognition of infants using a non-contact fingerprint scanner specifically designed to capture infant prints. They have described in detail multiple iterations of creating and testing the technology both in lab and operational environment. In general, this is a good contribution focused on solving a fundamental problem in the biometrics research community. However, there are some key aspects authors should consider to further improve the manuscript:

1. The use of term 'recognition' would be better compared to identification because it would encompass both verification (1:1 comparison) and identification (1:N comparison) scenarios.
2. A critical citation (Infant Prints: Fingerprints for reducing Infant Mortality by Engelsma *et al*. ^1^) is missing. In that report, the authors target recognition of 0-3 months old infants and show promising results. Please cite and rewrite portions of manuscript accordingly.
3. The authors use human-centered design principles towards solving the problem and comment that 'infants are uncooperative and the device design and collection protocol must be consistent with how babies, and often caregivers, behave, not how technology functions best.' Question: why not optimize both?
4. There were some suggestions provided by the cited report by Jain *et al.* on how best to capture contact fingerprints. How do the suggestions there compare to what is suggested in the manuscript here?
5. The statement: "Likewise, physiological changes that increase skin integrity, including keratinization, collagen development, and component cross-linking continue to develop after birth, and in some cases may not stabilize sufficiently for contact-based fingerprinting until over one year of age". Is this just an assertion or scientifically proven? Please give appropriate citation.
6. The authors use Verifinger to extract templates from fingerprints. Which template format is used? It is said it is standard but by default Verifinger uses a proprietary template that uses other features in addition to standard minutiae features. If minutiae only template is used for interoperability, there must be some loss in performance. Please provide details.
7. While a very detailed description of the scanner and its capabilities is provided, please provide details of initial recognition experiments (verification in terms of TAR @FAR=0.1% for example, and identification in terms of rank-1 accuracy) as well as comparison with contact scanners in terms of recognition performance.

I confirm that I have read this submission and believe that I have an appropriate level of expertise to confirm that it is of an acceptable scientific standard, however I have significant reservations, as outlined above.

## References

[1] : Infant-Prints: Fingerprints for Reducing Infant Mortality. *arXiv e-prints*.2019; Reference source

# Response

**Published:** 2019-10-08

**Authors:** Eliah Aronoff-Spencer

Thank you to reviewer 2 for your comments. Please see notes below. 

1. “The use of term 'recognition' would be better compared to identification because it would encompass both verification (1:1 comparison) and identification (1:N comparison) scenarios.”

- We appreciate the comment and have changed the title in response.

2. “A critical citation (Infant Prints: Fingerprints for reducing Infant Mortality by Engelsma *et al*. 1) is missing. In that report, the authors target recognition of 0-3 months old infants and show promising results. Please cite and rewrite portions of manuscript accordingly.”

- This is an excellent recent article that had not been published at the time of our paper. We have also cited this article in a recently submitted follow up to this report. We do note the results are similar to prior promising work by Jain’s group using a high resolution NEC sensor.

3. “The authors use human-centered design principles towards solving the problem and comment that 'infants are uncooperative and the device design and collection protocol must be consistent with how babies, and often caregivers, behave, not how technology functions best.' Question: why not optimize both?”

- We appreciate this comment. The key point is that we must design for and with the user and use the best technology that works within a users (here this might be a biometrician, a caregiver as well as the child) constraints. Optimizing technology can be misunderstood as optimizing existing approaches, this might include improving a contact fingerprint sensor by increasing the resolution rather than the approach, for instance. Likewise, iris scanners are technically quite feasible for infant biometrics, yet these can show significant failure modes due to infant behavior and caregiver attitudes. Sometimes the best technology can’t be used for non-technical reasons. Here we propose that infant fingerprinting is an optimal approach, yet contact sensors may be a fundamental failure mode with very young infants. This is due to a combination of infant interaction and developmental stage of their friction ridges.Thus we changed our approach, moving to non-contact and considering ergonomics, and we optimized the technology within the constraints of this new interaction space.

4. “There were some suggestions provided by the cited report by Jain et al. on how best to capture contact fingerprints. How do the suggestions there compare to what is suggested in the manuscript here?”

- Reported methods for cleaning infant fingers, assuring there is not debris, peeling skin etc, working with infant and caregiver,  are relevant for contact or non-contact approaches. One should note however, that no amount of skin preparation was able to overcome deformations of the print created by contact methods in the youngest subjects. 

5. "The statement: "Likewise, physiological changes that increase skin integrity, including keratinization, collagen development, and component cross-linking continue to develop after birth, and in some cases may not stabilize sufficiently for contact-based fingerprinting until over one year of age". Is this just an assertion or scientifically proven? Please give appropriate citation."

1. This is referenced here: 1. Babler WJ: Embryologic development of epidermal ridges and their configurations. Birth Defects Orig Artic Ser. 1991; 27(2): 95–112. PubMedAbstract
2. Additional reference worth noting: Lane, A. Human fetal skin development. Pediatr. Dermatol. (1986).

6. “The authors use Verifinger to extract templates from fingerprints. Which template format is used? It is said it is standard but by default Verifinger uses a proprietary template that uses other features in addition to standard minutiae features. If minutiae only template is used for interoperability, there must be some loss in performance. Please provide details.”

- The reviewer is correct. For these experiments we use the default Verifinger templates. It is however possible to export interoperable templates which we have verified, but may not be relevant to this report.

7. “While a very detailed description of the scanner and its capabilities is provided, please provide details of initial recognition experiments (verification in terms of TAR @FAR=0.1% for example, and identification in terms of rank-1 accuracy) as well as comparison with contact scanners in terms of recognition performance.”

- Full description of our clinical trial will be discussed in other publications. However, we have updated this paper to discuss our data analysis optimization and the first pass analysis we have performed on a limited dataset.

# Peer review: Reviewer response for version 1

**DOI:** 10.21956/gatesopenres.14013.r27440

**Published:** 2019-07-09

**Authors:** Arathi Arakala

**Affiliations:** 1 School of Mathematical and Geospatial Sciences, RMIT University, Melbourne, Australia

This is a very well written article on a possible solution to the infant biometric problem.

 The authors identified the key issues with capturing infant biometrics and walked us through their thought process in evaluating different biometric modalities as candidates. They narrowed down on fingerprints and identified the key challenges in using this for infant verification. Some of the challenges they highlighted were the uncooperative nature of the infants with the practitioner when capturing the biometric, skin peeling in the days after birth, high malleability of skin leading to extreme non linear distortions in capture with contact and very low inter-ridge spacing in the early days after birth.

 The authors then went on to describe step by step how they overcame each challenge by focussing on the natural interaction of the infant with the technology and using a cycle of developing, testing, evaluating and recalibrating their design.

 My only feedback would be to substantiate their claim that the initial 100 fingerprints captured had 100% verification even when the prints were taken from new borns. This could be in the form of a histogram of match scores, examples of comparisons that did match but with scores on the lower end of the spectrum and higher end of the spectrum. Also some information on the time difference between matched fingerprints. Were they taken from the infant on the same day, were they taken a few days or months apart? This would give us an idea how the image processing adapts to longitudinal changes in the fingerprint.

 Overall, a very enjoyable and informative read.

I confirm that I have read this submission and believe that I have an appropriate level of expertise to confirm that it is of an acceptable scientific standard.

# Response

**Published:** 2019-10-08

**Authors:** Eliah Aronoff-Spencer

We very much appreciate this reviewers feedback. We have taken excerpts from your suggestions and pasted below with our responses. 

1. “My only feedback would be to substantiate their claim that the initial 100 fingerprints captured had 100% verification even when the prints were taken from newborns. This could be in the form of a histogram of match scores, examples of comparisons that did match but with scores on the lower end of the spectrum and higher end of the spectrum. “

- We have updated the paper with preliminary performance data. 

2. “Also some information on the time difference between matched fingerprints. Were they taken from the infant on the same day, were they taken a few days or months apart? This would give us an idea how the image processing adapts to longitudinal changes in the fingerprint.”

- We have updated the report. Now we note the age of enrollment (0->30 days) as well as time between visits (ave, max, min in this dataset, up to 2 years in the full dataset).
